# *In silico* evaluation of the compounds of the ayurvedic drug, AYUSH-64, for the action against the SARS-CoV-2 main protease

**DOI:** 10.1016/j.jaim.2021.02.004

**Published:** 2021-02-25

**Authors:** Thrigulla Saketh Ram, Manne Munikumar, Vankudavath Naik Raju, Parasannanavar Devaraj, Naveen Kumar Boiroju, Rajkumar Hemalatha, P.V.V. Prasad, Manohar Gundeti, Brijesh S. Sisodia, Sharad Pawar, G.P. Prasad, Mukesh Chincholikar, Sumeet Goel, Anupam Mangal, Sudesh Gaidhani, N. Srikanth, K.S. Dhiman

**Affiliations:** a CCRAS-National Institute of Indian Medical Heritage, Revenue Board Colony, Gaddiannaram, Hyderabad, 500036, Telangana State, India; b NIN-TATA Centre for Excellence in Public Health Nutrition, ICMR-National Institute of Nutrition, Hyderabad, 500007, Telangana State, India; c Nutrition Information, Communication & Health Education (NICHE), ICMR-National Institute of Nutrition, Hyderabad, 500007, Telangana State, India; d ICMR-National Institute of Nutrition, Hyderabad, 500007, Telangana State, India; e CCRAS-Raja Ramdeo Anandilal Podar (RRAP) Central Ayurveda Research Institute for Cancer, Mumbai, India; f CCRAS-Regional Ayurveda Research Institute for Drug Development, Gwalior, India; g CCRAS-Regional Ayurveda Institute for Fundamental Research, Pune, India; h Central Council for Research in Ayurvedic Sciences, New Delhi, India

**Keywords:** COVID-19, SARS-CoV-2, AYUSH-64, Main protease, Molecular docking, Dynamics simulations

## Abstract

**Background:**

Outbreak of Corona Virus Disease in late 2019 (COVID-19) has become a pandemic global Public health emergency. Since there is no approved anti-viral drug or vaccine declared for the disease and investigating existing drugs against the COVID-19.

**Objective:**

AYUSH-64 is an Ayurvedic formulation, developed and patented by Central Council of Research in Ayurvedic Sciences, India, has been in clinical use as anti-malarial, anti-inflammatory, anti-pyretic drug for few decades. Thus, the present study was undertaken to evaluate AYUSH-64 compounds available in this drug against Severe Acute Respiratory Syndrome-Corona Virus (SARS-CoV-2) Main Protease (M^pro^; PDB ID: 6LU7) via *in silico* techniques.

**Materials and methods:**

Different molecular docking software's of Discovery studio and Auto Dock Vina were used for drugs from selected AYUSH-64 compounds against SARS-CoV-2. We also conducted 100 ns period of molecular dynamics simulations with Desmond and further MM/GBSA for the best complex of AYUSH-64 with M^pro^ of SARS-CoV-2.

**Results:**

Among 36 compounds of four ingredients of AYUSH-64 screened, 35 observed to exhibits good binding energies than the published positive co–crystal compound of N3 pepetide. The best affinity and interactions of Akuammicine N-Oxide (from *Alstonia scholaris*) towards the M^pro^ with binding energy (AutoDock Vina) of −8.4 kcal/mol and Discovery studio of Libdock score of 147.92 kcal/mol. Further, molecular dynamics simulations with MM-GBSA were also performed for M^pro^– Akuammicine N-Oxide docked complex to identify the stability, specific interaction between the enzyme and the ligand. Akuammicine N-Oxide is strongly formed h-bonds with crucial M^pro^ residues, Cys145, and His164.

**Conclusion:**

The results provide lead that, the presence of M^pro^– Akuammicine N-Oxide with highest M^pro^ binding energy along with other 34 chemical compounds having similar activity as part of AYUSH-64 make it a suitable candidate for repurposing to management of COVID-19 by further validating through experimental, clinical studies.

## Highlights

• Main protease (M^pro^) is a molecular drug target for the 2019-nCoV of epidemic disease of COVID-19.
• Docking strategies implemented to identify AUSH-64 having dual role as immunomodualtor and inhibition against M^pro^ of SARS-CoV-2.
• Molecular dynamics stability analysis revealed that 2019-nCoV M^pro^ – Akuammicine N-Oxide is stable.
• Akuammicine N-Oxide may represent potential treatment options against M^pro^ of 2019-nCoV.

## Graphical abstract

**Figure:**
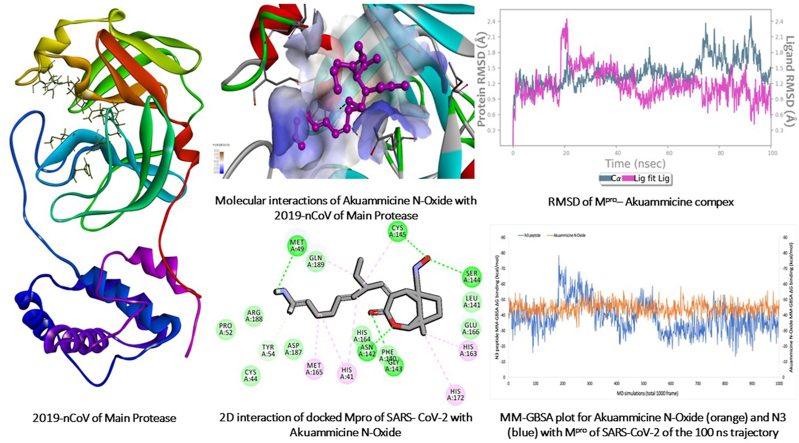

## 1 Introduction

The outbreak of corona virus disease (now known as COVID-19) in late 2019 in Wuhan, China led to a global public health emergency that currently affects more than 200 countries worldwide. Coronavirus, crown-like spikes positive-sense ribonucleic acid (RNA) from Coronaviridae family. Over the past two decades, severe acute respiratory syndrome coronavirus (SARS-CoV) and Middle East respiratory syndrome coronavirus (MERS-CoV) led to pandemic in 2002 and 2012 resulted in a 10% and 37% mortality rate respectively [1]. In December 2019, a new coronavirus of probable bat origin caused an outbreak of human pulmonary disease in Wuhan, China, that quickly spread throughout the world [2,3]. The causative agent initially named as 2019-novel coronavirus (2019-nCoV) by world health organisation (WHO). As per name recommendation from International Committee of Corona virus Study Group (ICCSG), the virus named as Severe Acute Respiratory Syndrome Coronavirus-2 (SARS-CoV-2), its RNA genome shares 82.30% and similar pathogenesis (host) of its identity with SARS coronavirus (SARS-CoV) [1,4]. Ultimately, the disease became known as corona virus disease 2019 (COVID-19) (WHO, 2020). On January 30, 2020, WHO officially declared the COVID-19 epidemic a public health emergency of international concern, and on March 11, 2020, the outbreak was confirmed a pandemic (WHO, 2020). The SARS-CoV and SARS-CoV-2 shares the similar pathogenesis (host) due to similarities in their genome [1].

Though several drug targets of coronaviruses have been identified, the main protease (M^pro^), also known as 3-chymotrypsin-like protease (3CL^pro^), has emerged as the best-described drug target [5]. The M^pro^ processes the large polypeptide, translated from the viral RNA, specifically at 11 splicing regions mostly Leu to Gln (Ser, Ala and Gly). These significant splicing regions non-homologous to human. Inhibiting enzyme activity might results in blocking viral pathogenesis, making it an attractive drug target for SARS-CoV-2. Further, a mechanism-based peptide-like inhibitor (N3) has also been identified via computer-aided drug design, and then crystal structure of the M^pro^ of SARS-CoV-2 in complex with this compound has been determined [6].

Given the lack of any validated, approved therapeutic intervention, such as a drug or vaccine, the COVID-19 pandemic continues to spread around the world. In such circumstances, the concept of drug repositioning might be a cost-effective, time-efficient, and less labour-intensive option for development of possible therapeutic and/or prophylactic lead candidate(s) from existing traditional and/or approved drugs [7]. In comparison to synthetic inhibitors, plant based-drugs are known to have low toxicity and are much safer to use. Natural products, such as traditional medicines and plant-derived compounds (phytochemicals) are considered rich sources of promising antiviral drugs [8].

Natural products were the sources of approximately 44% of the approved antiviral drugs from 1981 to 2006 [9]. Recent studies have suggested that drug poisoning and severe to moderate adverse effects associated with hydroxychloroquine have been reported in diabetic and hypersensitive patients, in whom COVID-19 tends to affect severely. Further, hydroxychloroquine is also known for inhibition of pro-inflammatory cytokines which leads to Acute Respiratory Distress Syndrome (ARDS) [10]. Due to the severe adverse effects shown by these synthetic compounds, natural, non-synthetic, target specific drugs with minimal side effects are urgently required for prophylaxis and treatment of COVID-19 [11].

Plant compounds and natural phyto-molecules are most economical and ideal for exploration to develop drugs through the drug discovery process [12]. Recent studies via integrated computational approaches have highlighted the repurposing of approved and known drugs against SARS-CoV-2 drug targets, either individually or in combination, to combat the virulence of COVID-19. Several plant compounds have been reported, using *in silico* approaches, as potential inhibitors of the M^pro^ of SARS-CoV-2. Mimicked drugs of bictegravir, doultegravir, paritaprevir, and raltegravir have been studied against 3CLpro and 2′-OMTase through *in silico* approaches [13,14]. Another study reported darunavir, remdesivir, and saquinavir, the natural compounds derivatives from coumarine and flavone which inhibits 3CLpro [[13], [14], [15]]. In another *in silico* study also shown that, *Andrographis paniculata's* compound Andrographolide, inhibits M^pro^ of SARS-COV-2. Its good solubility, pharmacodynamic properties, target accuracy, and adherence to Lipinski's rule of five were also predicted using computational tools [11].

The potent plant compounds nelfinavir, lopinavir, kaempferol, quercetin, luteolin-7-glucoside, demethoxycurcumin, naringenin, apigenin-7-glucoside, oleuropein, curcumin, catechin, and epicatechin-gallate, have been shown to inhibit the M^pro^ [12]. At the same time, several potential anti-SARS-CoV-2 lead compounds have been revealed in the medicinal plant library of traditional Chinese medicine compounds [16]. Another study identified oolonghomobisflavan-A from the tea plant as a potential inhibitor against M^pro^ of SARS-CoV-2 [17]. In addition, a promising drug candidate from plant sources, bonducellpin D, has been reported to exhibit broad-spectrum inhibition potential against both the SARS-CoV M^pro^ and the MERS-CoV M^pro^ [18].

AYUSH-64 is a poly-herbal compound developed by the Central Council for Research in Ayurvedic Sciences, the main organization for research and development in Ayurveda in India under the Ministry of AYUSH, Government of India. It consists of four ingredients viz., *Saptaparna* (*Alstonia scholaris* R. Br.) bark aqueous extract 100 mg, *Katuki* (*Picrorhiza kurroa* Royle ex. Benth) root extract 100 mg, *Kiratatikta* (*Swertia Chirata* Pexbex. Karst) whole-plant extract 100 mg, and *Kuberaksha* (*Caesalpinia crista* L.) seed powder 200 mg [19].

To summarize the information provided in Table 1, it is understood that all four ingredients of AYUSH-64 are characterized by *tikta rasa* (bitter taste) and therefore *amapachak* (able to digest the *Ama* or undigested form of food) and hence act as *Jvaraghna* (anti-pyretic). The combined effects of these herbs are *jwarahara* (able to relieve fever)*, sannipata jwarahara* (able to relieve intermittent fevers), *krimihara* (wormicidal), *jantuhara* (anthelmintic/antimicrobial/antiviral), and *shothahara* (anti-inflammatory), rendering this compound a potent combination against conditions such as Influenza Like Illnesses having the symptoms of cough, cold, headache and fever. Several compounds found in the drug have been reported to exhibit several pharmacological activities including anti-malarial, anti-viral, anti-inflammatory, immunomodulatory etc. (Table 2).

**Table 1:** Ayurvedic Pharmacological profile of the plants in AYUSH-64.

Name of the plant	*Rasa* (Taste)	*Guna* (Property/Quality)	*Virya* (Potency)	*Vipaka* (bio-transformed *rasa*)	*Karma* (Pharmacological actions)
*Saptaparna* [[40], [41], [42]] (*Alstonia scholaris* R. Br.)	*Tikta (bitter), Kashaya (astringent)*	*Sara, Ushna (hot), Snigdha (oleus), Deepan (kindling), Laghu (light)*	*Ushna (hot)*	*Katu (pungent)*	*Shoolahara* (relieves pain)*, Gulmahara* (relieves bloating)*, Krimihara* (wormicidal)*, Hrudya* (cardiac tonic)*, Shwashara* (useful in asthma)*, Vranahara* (wound healer)*, Asradoshahara* (useful in blood related diseases)*, Jantuhara* (antihelminthic)*, Tridoshaghna* (pacifies *kapha, pitta* and *vata*), *Kasahara* (useful in Bronchitis) *Kushthaghna* (useful in skin disorders), *Jvaraghna* (Antipyretic), *Sarakta Pravahikahar* (useful in bloody dysentery), *Vataraktahara* (*useful in Gout*), *Grahanihara* (useful in IBS like conditions) *Udardaprashaman* (relieves urticaria), *Dantakrimihara* (useful in dental caries), *Vishaghna* (Antitoxic)
*Katuki* [41,42] (*Picrorhiza kurroa* Royle ex. Benth)	*Tikta (bitter)*	*Laghu (light), Ruksha (dry)*	*Shita (cold)*	*Katu (pungent)*	*Bhedana* (causes purgation)*, Deepana* (improves digestion)*, Hrudya* (cardiac tonic)*, Jwarahara* (useful in fevers)*, Vishamjvaranashini* (useful in recurrent fevers) *Kaphapittahara* (pacifies *Kapha* and *pitta), Pramehaghna* (useful in urinary disorders/diabetes)*, Shwasa-Kaasaghna* (useful in asthma, cough)*, Dahaghna* (relieves burning sensation)*, Kushthaghna* (useful in skin disorders), *Lekhana* (therapeutic scrapping), *Krimihara* (wormicidal), *Arochakaghna* (useful in tastelessness) *Asrajit* (useful in blood disorders)*, Stanyashodhan* (improves quality of breast milk) *Hikkanigrahana* (relieves hiccups)
*Kiratatikta* [41,42] (*Swertia Chirata* Pexbex. Karst)	*Tikta (bitter)*	*Laghu (light), Ruksha (dry)*	*Shita (cold)*	*Katu (pungent)*	*Sannipatajwarahara* (useful in chronic and recurrent fevers)*, Shwasahara* (useful in asthma)*, Kaphapittahara* (pacifies *Kapha* and *pitta), Asradoshahara* (useful in blood related diseases)*, Dahashamana* (relieves burning sensation)*, Kasahara* (useful in cough)*, Shothahara* (relieves inflammation)*, Trishnashamaka* (relieves thirst)*, Kushthahara (*useful in skin disorders)*, Vranahara* (wound healer)*, Krimihara* (wormicidal), *Stanyashodhan* (improves quality of breast milk), *Jvaraghna* (Antipyretic), *Raktapittahara* (useful in bleeding disorders)
*Kuberaksha* [43] (*Caesalpinia crista* L.)	*Katu (pungent), Tikta (bitter), Kashaya (astringent)*	*Laghu (light) Ruksha (dry)*	*Ushna (hot)*	*Katu (pungent)*	*Kaphavatahara* (pacifies *Kapha* and *vata), Deepana* (improves digestion)*, Shoolaghna* (relieves pain)*, Gulmanaashaka* (relieves bloating)*, Kriminashaka* (wormicidal)*, Kushthanaashaka (*useful in skin disorders)*, Pramehajit* (useful in urinary disorders/diabetes)*, Pittarshanaashaka* (useful in haemorrhoids)*, Vamihara* (antiemetic), *Sramsana* (laxative), *Shothahara* (anti-inflammatory), *Vranaropana* (wound healer)*,Yakrutplihaghni* (useful in Liver & spleen related disorders), *Vataghna* (pacifies vata), *Kaphaja Shlipadahara* (useful in filariasis)

**Table 2:** The pharmacological activity of AYUSH-64 by ingredient.

S.No	Name of the plant	Chemical Compound(s) & pharmacological activities.
1.	*Saptaparna (Alstonia scholaris* R. Br.)	Methanol extracted from bark of *Alstonia scholaris* Found to be more promising. Nevertheless, the observed antiplasmodium activity of *Alstonia scholaris* was less pronounced as compared to *Alstonia macrophylla* [44]. *Alstonia macrophylla*, which is seen in India, generally adultrated for *Alstonia sholaris* [44]. Four new alkaloids*,* alstiphyllanines A-D (1–4), were isolated from *Alstonia macrophylla*, and their structures were determined by MS and 2D NMR analyses. Alkaloids 1–4 showed moderate anti-plasmodial activity against *Plasmodium falciparum* and vasorelaxant activity against phenylephrine-induced contraction of isolated rat aorta. Other Studies: 1. Inhibited the carrageenan-induced inflammation in the rat paw oedema study model [45]. 2. Controls Malarial fever by virtue of its strong schizonticidal activity [46]. 3. Anti- HSW and anti-adenovirus activity of indole alkaloids from leaves of Alstonia scholaris, with significant inhibitory activity against herpes simplex virus (HSV) and adenovirus [47]. 4. Enhances DNA repair capacity [48]. 5. In vitro tests, alkaloids exhibited inhibition of inflammatorymediators (COX-1, COX-2 and 5-LOX), which is accordant with results on animal model [49]. 6. Potent antiplasmodial activity against P. falciparum [50,51].
2.	*Katuki* (*Picrorhiza kurroa* Royle ex. Benth)	1. Anti-inflammatory effect by l3-adrenergic blockade [52]. 2. Stimulates the cell-mediated and humoral components of the immune system as well as phagocytosis in experimental animals [53,54]. 3. Improves the immune system by increasing the proliferation of lymphocytes and cytokine levels (IL-4 and IFN-gamma) in serum, in HA titre, DTH, PFC, phagocytic index and CD4/CD8 population [55]. 4. Another study demonstrates the antioxidant and free radical scavenging activity of the leaf extract of same plant [56]. 5. Inhibits the growth of *Plasmodium falciparum* Significantly [[57], [58], [59]].
3*.*	*Kiratatikta (Swertia Chirata* Pexbex. Karst*)*	The drug shows promising effect due to itsantipyretic (Bhargava, 2009), and antimalarial activity [58]. 1. Anti-protozoal activity:A MeOH extract of Swertia chirata found to inhibit the catalytic activity of topoisomerase I of *Leishmania donovani* was subjected to fractionation to yield three secoiridoid glycosides: amarogentin (a), amaroswerin (b), and sweroside (c). Amarogentin is a potent inhibitor of type I DNA topoisomerase from Leishmania and exerts its effect by interaction with the enzyme, preventing binary complex formation. Other Studies: 2. Inhibits the expression of viral protein R (an attractive target for HIV disease) in HeLa cells harbouring the TREx plasmid encoding full-length Vpr (TREx-HeLa-Vpr cells) [60]. 3. Inhibited HSV-1, plaque formation at more than 70% level and viral dissemination [61]. 4. Suppressive effects on inflammatory mediators by blocking the expression of COX-2 and phosphorylation of Akt, IKK-β, MAPK and NF-κB, activation in LPS-stimulated macrophages [62]. 5. A study on experimental arthritis in rats suggested amelioration of oxidative and inflammatory stress, which implies the immunomodulatory effect of leavesof the above said plant. Post treatment with the leaves of the said plant, the animal subjects showed marked reduction in inflammation as well as arthritic changes [63].
4*.*	*Kuberaksha* (*Caesalpinia crista* L.)	1. The CH_2_Cl_2_ extract from the seed kernels of *Caesalpinia crista*, which exhibited a promising antimalarial activity against *Plasmodium berghei* infected mice *in vivo*, which was examined and resulted in the isolation of seven new furanocassane-type diterpenes [caesalpinins C-G (1–5) and norcaesalpinins D and E (6, 7)] together with norcaesalpinins A-C (8–10) and 11 known compounds (norcaesalpinins A-C, 2-acetoxy-3-deacetoxycaesaldekarin e, caesalmin B, caesaldekarin e, caesalpin F, 14(17)-dehydrocaesalpin F, 2-acetoxycaesaldekarin e, 7-acetoxybonducellpin C, and caesalmin G). Their structures were determined on the basis of spectroscopic analysis. The isolated diterpenes showed significant dose-dependent inhibitory effects on Plasmodium falciparum FCR-3/A2 growth in vitro. Their IC50 values ranged from 90 nM to 6.5 microM, and norcaesalpinin E (7) showed the most potent inhibitory activity (IC50, 90 nM). 2. Exhibits antimalarial [64] and hepatoprotective [65]activity Other studies: 3. Protection against red blood cell (RBC) haemolysis and DNA damage [66]. 4. Immuno-stimulatory: increase in hemagglutinating antibody titre and a change in delayed-type hypersensitivity [26]. 5. Two weeks after challenge with *Pseudomonas aeruginosa*, the Caesalpinia treated animals showed a significant bacterial clearance from the lungs, with less severe incidence of lung abscess [67]. 6. Exhibited activity against the vaccinia virus [68]. 7. *in vivo* experimental study, neutrophil adhesion test, hemagglutinating antibody (HA) titre, delayed-type hypersensitivity (DTH) response, phagocytic activity and cyclophosphamide-induced myelosuppression were demonstrated to be positively activated pointing towards a promise in immunomodulation [26].

Various pre-clinical studies were performed on AYUSH-64 to study its safety and efficacy aspects (Table 3). The major studies, namely anti-malarial property in albino mice, found safe and non-toxic in a dose of 500 mg/kg of body weight for 12 weeks (Anonymous, 1987) and potential antiviral activity of Chirakin (marketed name of AYUSH-64 by Zandu) against Chikungunya virus were investigated at the department of Molecular Virology Laboratory, Rajiv Gandhi Centre for Biotechnology, Thiruvananthapuram, by protection and plaque reduction assay. The efficacy of the compound was expressed in terms of activity index and selectivity index in protection assay and Vero cells were infected with Chikungunya Virus (CHIKV) and treated with an optimum concentration of Chirakin (25 μg/mL). Chirakin treated cells and the reduction in virus yield was determined by plaque assay in Vero cells. Chirakin shows antiviral activity against Chikungunya virus in this study. Its activity was found better than ribavirin in protection assays, where as in plaque reduction assays, both perform almost equally with a 2 log_10_ reduction in virus number (CK Katiyar. Technical note on Chirakin Tab –Unpublished report).

**Table 3:** Preclinical pharmacological and toxicological/safety studies of AYUSH-64 and its ingredients.

S. No	Preclinical pharmacological and toxicological/safety studies
1.	In albino mice, oral administration of AYUSH-64 at doses of 250–750 mg/kg for five days exhibited significant anti-malarial property [67].
2.	The experimental studies of AYUSH-64 have shown that it was safe and non-toxic in a dose of 500 mg/kg of body weight for 12 weeks [67].

Attempts have also been made to clinically evaluate the compound for management of Microfilariasis [20,21] effectiveness against *Plasmodium vivax* in ring stage than in gamete stage and also the mixed infection cases of *P. vivax* [22] decline in infectivity rate of malaria, double-blind study for comparative efficacy in comparison with chloroquine/primaquine in *P. vivax* malaria and lead candidate in the management of influenza like illnesses [23] (Table 4). Overall, the compound is considered to be safe and is prescribed widely by Ayurvedic experts for the effective management of malaria, joint pains, fever, and influenza like illnesses. In this *in silico* study*,* 36 major compounds were analysed which are four ingredients of AYUSH-64 drug might be potent inhibitors against M^pro^ of SARS-CoV-2 observed via two different docking strategies of AutoDock Vina and Discovery studio molecular docking tools.

**Table 4:** Clinical studies of AYUSH-64.

S.No	Clinical studies of AYUSH-64
1	Clinically effective in cases of microfilariasis [20,21].
2	81% curative effect on *Plasmodium vivax*, drug is more effective in ring stage than in Gamete stage. In the cases of mixed infection of *P.vivax* and *P.falciparum* the curative effect was found to be 75% after longer therapy [69].
3	Good effect in the management of microfilaraemia [70].
4	In a study group of 4500 participants, AYUSH-64 was observed to be safe and nontoxic with good anti-malarial activity and also a major decline in the infectivity rate was noted by administering AYUSH-64 along with anti- mosquito measures, in various types of fevers [70].
5	A double-blind study demonstrated comparative efficacy of AYUSH-64 to the chloroquine/primaquine as the standard modern control in sixty cases of *P.vivax* malaria [70].
6	In a prospective, open-label, nonrandomized, single group, single-centre pilot study with pre-test and post-test design one-week intervention of ‘AYUSH-64’ in a dose of 3 g/day effectively helped to recover from Influenza Like Illnesses, and also the quicker return to normal life with reduced frequency of usage of acetaminophen/antihistaminic. No adverse effects were found during the study [71]
7	A pilot study done on 112 participants produced 97.14% result with prophylactic treatment in P.F. Malaria [72].

## 2 Materials and methods

In the drug discovery process for COVID-19 crisis, the development of novel drugs with potential interactions with therapeutic targets is of primal importance. Conventionally, promising-lead or drug identification is achieved by traditional or experimental high-throughput screening (HTS), which is time-consuming and cost-effective [24]. In contrast to the traditional drug discovery method (classical or forward pharmacology), the rational drug design is noted as efficient and economical [25]. In these contrasts, approach to identifying therapeutics is to repurpose approved compounds developed for other uses, by taking advantage of existing detailed information on human pharmacology and toxicology to enable rapid clinical trials and regulatory quick review. Furthermore, *in silico* drug design method is also known as reverse pharmacology, as the initial step is to identify promising drug targets (proteins/enzymes), after which they are used for screening of novel small-molecule inhibitors for the candidates [14,[26], [27], [28]]. In the present study of *in silico* drug design method, molecular docking approach was used for SARS-CoV-2 M^pro^ and 36 AYUSH-64 compounds, to screen for the novel inhibitors by two different docking strategies/algorithms, LibDock protocol of BIOVIA Discovery Studio and AutoDock Vina.

### 2.1 M^pro^ structure preparation

The crystal structure of COVID-19 M^pro^ in complex with an peptide inhibitor N3 (n-[(5-methylisoxazol-3-yl)carbonyl]alanyl-l-valyl-n∼1∼-((1r,2z)-4-(benzyloxy)-4-oxo-1-{[(3r)-2oxopyrrolidin-3-yl]methyl}but-2-enyl)-l-leucinamide) peptide obtained from Protein Data Bank (PDB) [6]with the PDB ID of 6LU7 with crystal resolution 1.5 Ȧ for the molecular docking studies. Foremost, for the preparation of the protein Auto Dock Tool (ADT) used for removal of all HOH molecule, adding Kollman charges and polar hydrogen atoms, assigning hydrogen polarities. Further, Gasteiger charges were applied on prepared protein and protein structures file 6LU7. PDB converted to 6LU7. PDBQT [15].

In BIOVIA Discovery Studio, complex of M^pro^– N3 was prepared by removing all the water molecules, ligands and hetro-atoms from the complex. To satisfy the valency, hydrogen atoms were added to each atom. The M^pro^ structure was minimized by applying CHARMm vc41b1force field to remove the steric clashes between the atoms in order to get stable conformation. The 6LU7 amino acid residues, namely Thr24, Thr25, Thr26, Thr26, His41, Phe140, Leu141, Asn142, Gly143, Ser144, Cys145, His163, His164, Met165, Glu166, Pro168, His172, Arg188, Gln189, Thr190, Ala191, and Gln192 were found to be present within 4Ȧ region of N3 peptide binding site [6]. Among the listed 22 amino acids, Gly143, His163, His164, Glu166 (2), Gln189, and Thr190 residues were found to be formed eight hydrogen bonds with native N3 peptide having bond lengths of 2.87, 2.37, 2.8, 2.98, 2.83, 2.89, and 2.85 respectively. In both the docking strategies, the grid box was generated around the 4Ȧ region of N3 peptide in the M^pro^ of SARS-CoV-2 [15].

### 2.2 Ligand preparation

Selected 36 compounds of AYUSH-64 were obtained from the PubChem database, as 3D coordinates of structure-data file (SDF) format, and each compound was converted to PDBQT file format with the help of PyMol and ADT tool, generating an input compound/ligand file for docking study in AutoDock Vina. In BIOVIA Discovery studio, 225 maximum conformations were generated for selected 36 compounds, and separate isomer conformations were also created within the threshold of 20.0 kcal/mol relative energy. The CHARMm force field (fast and accurate) was applied to minimize the compounds with 1000 steps of steepest descent (SD) algorithm. The root-mean-square deviation (RMSD) with <1.0 Å were considered as duplicates, and the higher specified RMSD value will reduce the number of output ligand poses and RMS gradient 0.001 [15].

### 2.3 ADME and toxicity prediction

To predict the drug-likeness properties of selected 36 AYUSH-64 compounds, *in silico* absorption, distribution, metabolism, excretion, and toxicity (ADME/T) prediction was carried out. We explored the ADME/T properties of the 36 AYUSH-64 selected compounds using the ADME/T Protocol in the Discovery Studio software package (Accelrys, San Diego, CA, USA). These examinations were exclusively founded on the substance structure of the particle. Some of the parameters that were calculated included, ADME 2 Dimenctional Fast Polar Surface Area (ADME 2D FPSA), Atom-based Log P98 (ALogP98), Blood Brain Barrier (BBB), Cytochrome P4502D6 (CYP2D6), and Hepatotoxicity (HEPATOX).

### 2.4 Molecular docking

The prepared 36 AYUSH-64 compounds (ligands) were docked on an individual basis to the M^pro^ of SARS-CoV-2 with grid coordinates (grid center). The selected compounds were set flexible with conformation condition while interacting with the macromolecule of M^pro^ under rigid conditions of the protein. The prepared configuration file was run through ADT command line with PDBQT file as input with grid size of X: 25 × Y: 25 × Z: 25 points, grid centre set at X: 11.74, Y: 13.63 and Z: 70.63 dimensions having 1000 Å grid radius. By using AutoDock Vina scoring algorithm to measure Gibbs Free Energy (−ΔG kcal/mol) for selected AYUESH-64 compound binding affinities [29].

The LibDock protocol module of BIOVIA Discovery Studio was used for molecular docking studies of the 6LU7 and 36 AYUSH-64 compounds, the binding site is created with the same dimensions of the AutoDock Vina i.e. 4 Å radius of N3 peptide binding site. The LibDock protocol is a high-throughput algorithm developed by Diller and Merz, in 2001 [29], consisting the polar and a polar feature as “Hotspots.” Among the 36 compounds of AYUSH-64, which were passed the applied filters while ligand preparation process, subjected to dock with the prepared 6LU7 crystal structure. The prepared compounds were docked to analyses and compare interacting amino acids. The post-docking analyses of two different docking strategies were visualized for H-bond, hydrophobic molecular interactions and bond distances with 4 Å radii in the Discovery Studio v2020 in the M^pro^ of SARS-CoV-2. The top ranked molecular docked complex selected for molecular dynamics simulations using Desmond v2019-4 for 100 nano seconds (ns).

### 2.5 Molecular dynamics simulations

The first ranked docked molecular complex, AYUSH-64 with M^pro^ selected for carrying out the dynamic's simulations using Desmond v2019-4 protocol. Where initially the selected molecular docked complex atoms calibrated by the “Optimized Potentials for Liquid Simulation (OPLS-2005)” force field. Further, geometric chemical structure corrections in molecular docked complex are performed using “Protein Preparation Wizard (macro model)”. To put the molecular docked complex in solvent environment, Simple Point charge-HOH model was used with the cubic box dimensions 10 Å × 10 Å × 10 Å, and for maintaining the acid-base balance for the molecular docked complex solvent, electrical ions NaCL were added with 0.15 M [15,[30], [31], [32]]. To model the maximum possible atomic bond energy (covalent and non-covalent) flexibility of molecular docked complex, hybrid algorithm “Steepest Descent and the limited-memory Broyden-Fletcher-Goldfarb-Shanno” is used. To create the initial minimized condition model of dynamic particles, pressure, and temperature of the molecular docked complex, “Berendsen thermostat and barostat NPT ensemble” method is used for 5 ns.

To assert constant temperature at 300 K and pressure of 1 atm throughout the molecular dynamics’ simulation process, “Nose–Hoover thermostat algorithm [33]” and “Martyna-Tobias-Klein Barostat algorithm [34]” is used respectively. To affirm coulombic long-range atomic interactions in the molecular docked complex throughout the molecular dynamic simulations process “Smooth Particle Mesh Ewald” method is used. To maintain the accuracy and tolerance of long-range interactions with the smaller value of 1e-9 is set for accurate computational implemented by SHAKE algorithm. The final production run was carried out for 100 ns, and the trajectory sampling was done at an interval of 1.0 ps [15,30,31]. Subsequently, in the MD simulations study, the binding free energy (ΔG_bind_) for all simulated protein-ligand complexes were estimated by Desmond and Prime-molecular mechanics/generalized born surface area (MM-GBSA) method using thermal_mmgbsa.py script embedded in Schrödinger suite.

### 2.6 Prime MM-GBSA

The MM-GBSA is a suite of protocols that combines OPLS-2005 molecular mechanics energies (EMM), a Surface Generalized Born (SGB) solvation model for polar solvation (GSGB), and a nonpolar solvation term (GNP), composed of the nonpolar solvent accessible surface area and vdW interactions. The binding energy calculations are much accurate than the docking score [35]. The binding energy is calculated by the following equations [36,37].

(1) $$\Delta {\text{G}}_{\text{bind}}=\Delta_{\text{E}}+\Delta {\text{G}}_{\text{solv}}+\Delta {\text{G}}_{\text{SA}}$$

Where

(2) $$\Delta \text{E}={\text{E}}_{\text{complex}}-{\text{E}}_{\text{protein}}-{\text{E}}_{\text{ligand}}$$

Where *E*_complex_, *E*_protein_, and *E*_ligand_ are the minimized energies of the receptor–lead complex, receptor, and leads respectively

(3) $$\Delta {\text{G}}_{\text{solv}}={\text{G}}_{\text{solv}\;\left(\text{complex}\right)}-{\text{G}}_{\text{solv}\;\left(\text{protein}\right)}-{\text{G}}_{\text{solv}\;\left(\text{ligand}\right)}$$

Where *G*_solv(complex)_, *G*_solv(protein)_, and *G*_solv(ligand)_ are the solvation free energies of the complex, protein, and inhibitor, respectively

(4) $$\Delta {\text{G}}_{\text{SA}}={\text{G}}_{\text{SA}\;\left(\text{complex}\right)}{-\text{G}}_{\text{SA}\;\left(\text{protein}\right)}-{\text{G}}_{\text{SA}\;\left(\text{ligand}\right)}$$

Where *G*_SA (complex)_, *G*_SA (protein)_, and *G*_SA (ligand)_ are the surface area energies for the complex, protein and inhibitor, respectively.

The simulations were carried out using the GBSA continuum model in Prime [38]. The Prime uses a Surface Generalized Born (SGB) model employing a Gaussian surface instead of a vdW surface for better representation of the solvent-accessible surface area [35,39]. The binding free energy can be computed for all the trajectories or a set of frames within generated frames from MD simulations. Considering all frames, perfect (or imperfect) poses might have a crucial contribution in the binding free energy calculation, hence, each and every frame of the entire MD simulated trajectories were used, that is highly rigorous and more accurate approach to validate the docking and dynamics approaches in the study for calculating the binding free energy. Therefore, in the present study, all MD trajectory frames from start to end (∼1000 frames) were selected to estimate the ΔG_bind_ for proposed and native MD simulated complex.

## 3 Results

### 3.1 Validation of docking protocol

**Fig. 1:**
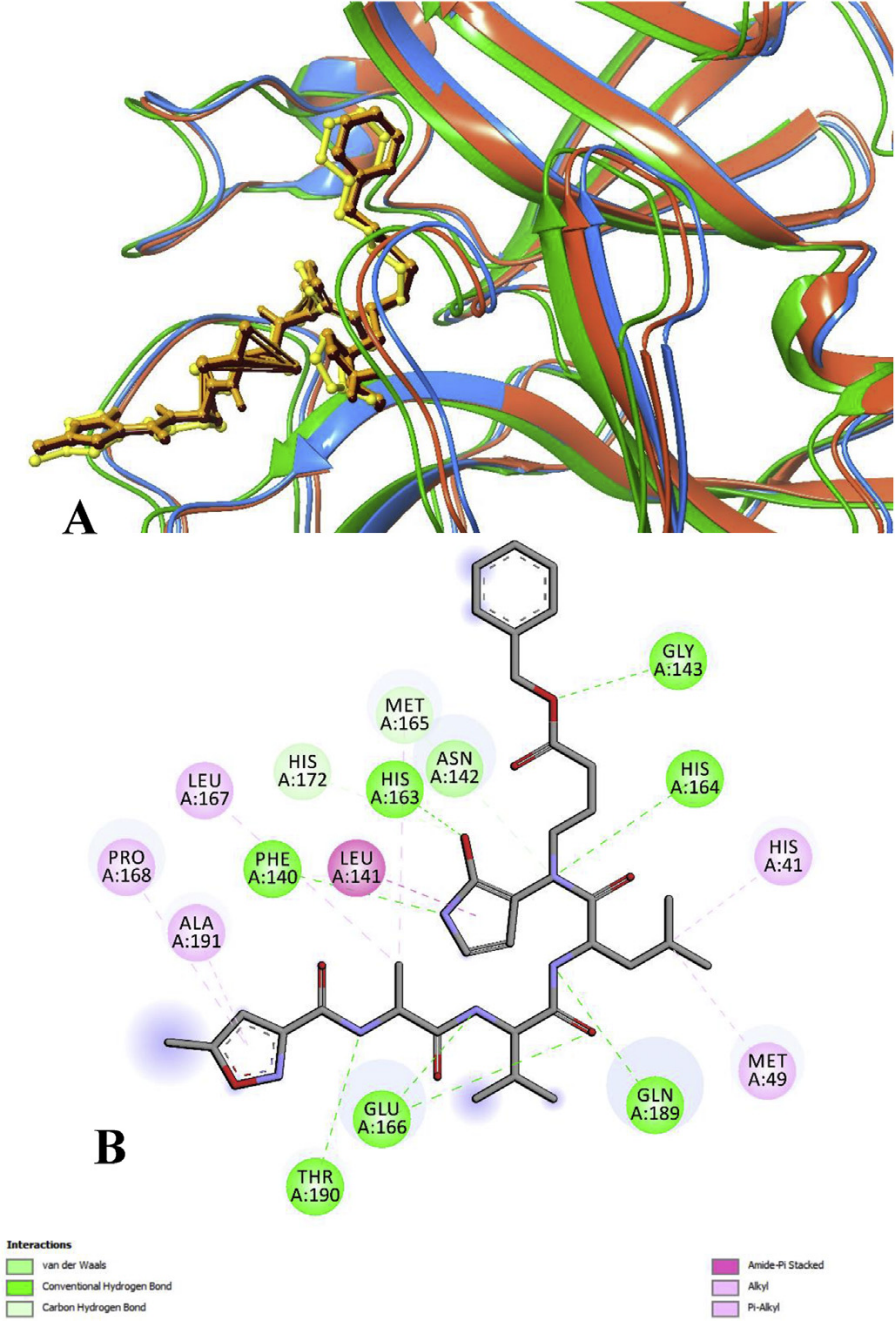
Superimposition of the docked M^pro^– N3 peptide with its X-ray crystal structure. **A)** Blue and yellow color indicates experimentally derived structure of M^pro^– N3 peptide, the docked complex of orange – brown and green – yellow orange is derived from Discovery studio and Autodock Vina docking approaches respectively. **B)** 2D interaction of experimental and docked M^pro^ of SARS- CoV-2.

Preferably, the distinguishing proof of the ideal docking and scoring blend will diminish the number of false positives and false negatives while ensuring optimal hit rates. It has accounted for enormous strategies for validating docking approach and scoring functions. Utilized strategy through pose selection whereby docking programs are exploited to re-dock into the target's active/binding site with a known conformation and orientation, ordinarily from known co–crystal structure. The approaches that can restore poses under a pre-selected RMSD esteem from the known compliance/orientation (1.5 or 2 Å relying upon ligand size) are considered having performed effectively (Fig. 1). Pose selection is then trailed by scoring and positioning to contemplate which of the accessible scoring capacities most precisely positions the stances regarding their RMSD esteems. In the present study, the different docking protocols of Discovery studio and AutoDock protocols are validated through, re-docked with known co–crystal structure of 6LU7 with N3 peptide (Fig. 1). The re-docked results of RMSD revealed that, the AutoDock vina determined with 1.25Ȧ and Discovery studio has 1.02Ȧ. Also, 2D atomic coordination of the re-docked complexes was supplanted experimentally derived structure. Thus, these docking protocols was viewed good enough for replicating the docking results similar to co–crystal structure and consequently can be applied for further molecular docking analysis.

### 3.2 Molecular interactions of M^pro^ with AYUSH-64

The SARS-CoV-2, M^pro^ plays a significant role in RNA translation and is essential for viral replication. The three-dimensional structure of M^pro^ (6LU7) is a monomer structure, consisting three major structural domains: domain I (residues 8–101), domain II (residues 102–184), which contribute an antiparallel β-barrel structure, and domain III (residues 201–303), which contains a five-fold anti-parallel α-helix cluster and is attached to long loop (residues 185 to 200) of domain II [6]. The amino acid of cysteine–Histidine dyadplays significant role in the maturation cleavage, which is located in the cleft between domains I and II the precursor M^pro^ [6]. Moreover, M^pro^ was non-homologous to human and it is an ideal anti-viral drug target for SARS-CoV-2 [15]. The co–crystal ligand of N3 peptide is located in the cleft region between domain I and II. The SARS-CoV-2 M^pro^ shares highly (∼99%) similarity and identity (100%) with binding site residues of SARS-CoV M^pro^ [15].

**Fig. 2:**
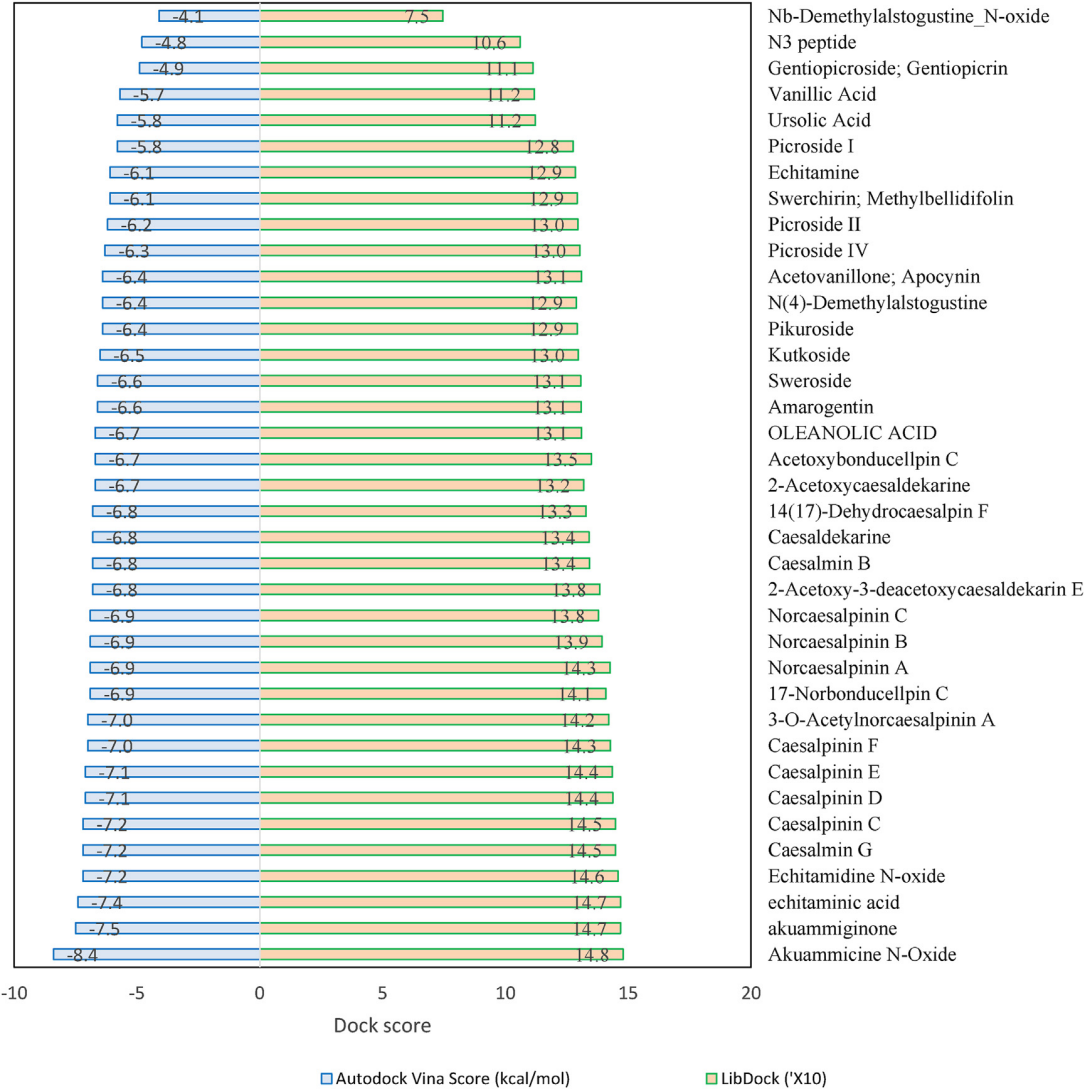
Molecular docking score of AYUSH-64 compounds. Among Akuammicine N-Oxide (green) has least docking sore of -8.4 kcal/mol and N3 peptide (red) has docking sore -4.1 kcal/mol.

**Fig. 3:**
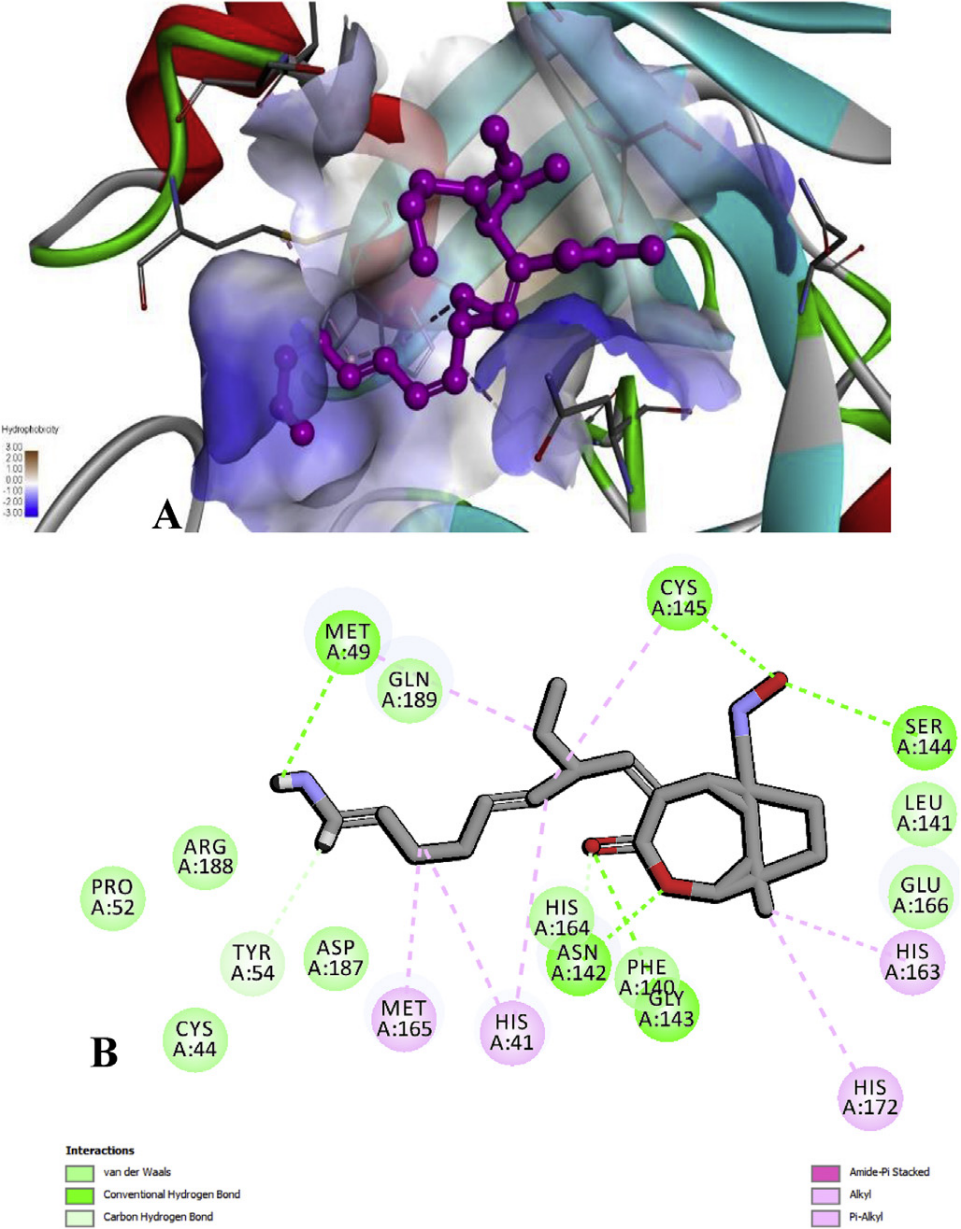
**A)** Molecular interactions of Akuammicine N-Oxide with 2019-nCoV of M^pro^. **B)** 2D interaction of docked M^pro^ of SARS- CoV-2 with Akuammicine N-Oxide.

In the present study, the selected 36 compounds of AYUSH-64 were substituted (shows a similar inhibitor-binding mode) within the cleft between domain I and II, found to be having good docking score than the native substrate of N3 peptide (Supplementary Table 1 and Fig. 1A). ADME/T properties prediction results are illustrated in Supplementary Table 2 for drug-likenes. Two docking strategies, Akuammicine N-Oxide compound has best docking score, binding affinity and good ADME/T properties than the other selected 35 AYUSH-64 compounds and reference substrate of N3 peptide (Supplementary Table 3 and Fig. 2). The side chain residues, His41 (2), Met49 (2), Tyr54, Asn 142 (2), Gly 143, Ser144, Cys145 (2), His163, Met165, and His172 formed 14 H-bonds with Akuammicine N-Oxide within 4 Å region of M^pro^ (Fig. 3). Along the side, Cys44, Pro52, Phe140, Leu141, Asn142, Arg188, Asp187, Gln189 were formed vdW interactions to the Akuammicine N-Oxide within 4 Å region in both docking strategies (Supplementary Table 2 and Fig. 3B). To summarize, Akuammicine N-Oxide compound reveal a similar inhibitory mechanism to occupy the substrate-binding pocket and block the enzyme activity of SARS-CoV-2 M^pro^. Further, the docked complex of M^pro^– Akuammicine N-Oxide and native M^pro^– N3 peptide complex was subjected to perform molecular dynamics simulations to check the stability at 100ns employing Desmond 2019-4.

### 3.3 Molecular dynamics simulations

**Fig. 4:**
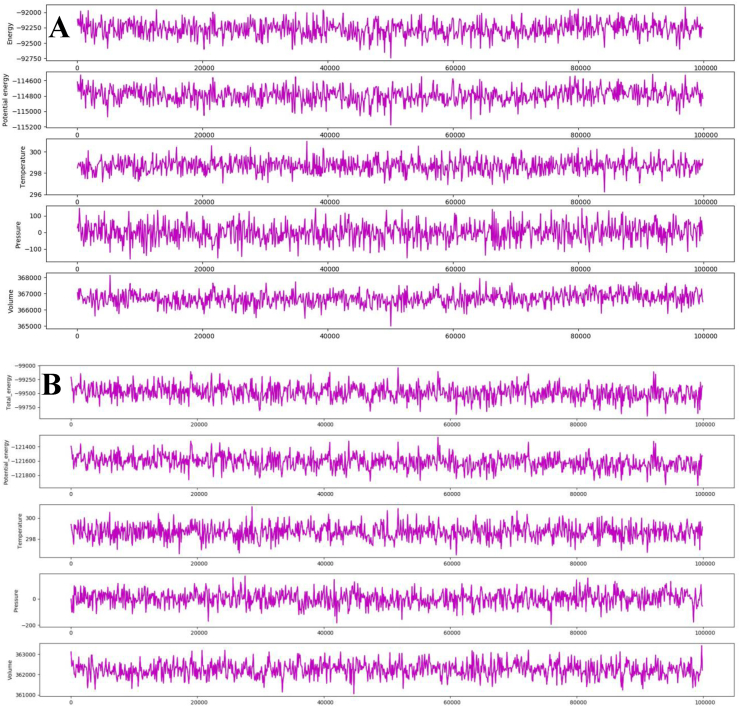
The stable total energy, potential energy, temperature, pressure and volume of molecular dynamics simulations at 100 ns period of 2019-nCoV M^pro^ with Akuammicine N-Oxide**(A)** and N3 peptide **(B)**.

**Fig. 5:**
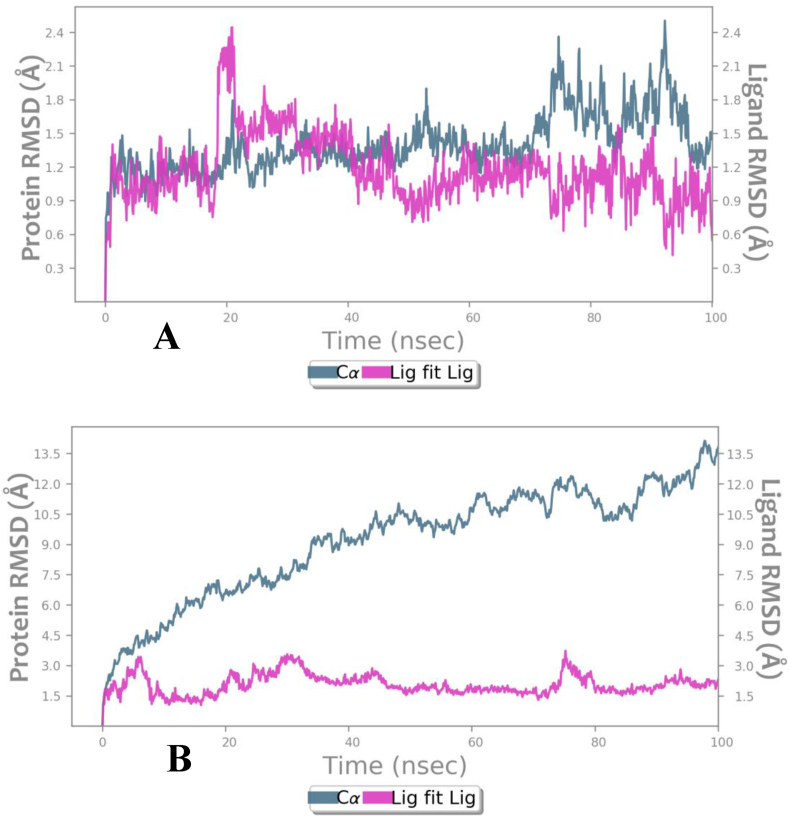
RMSD of protein Cα atoms of SARS-COv-2 M^pro^ (pink)and compounds (grayish green) during 100 ns of MD simulation. **(A)** M^pro^– Akuammicine compex **(B)** M^pro^– N-Oxide and N3 pepeide complex.

**Fig. 6:**
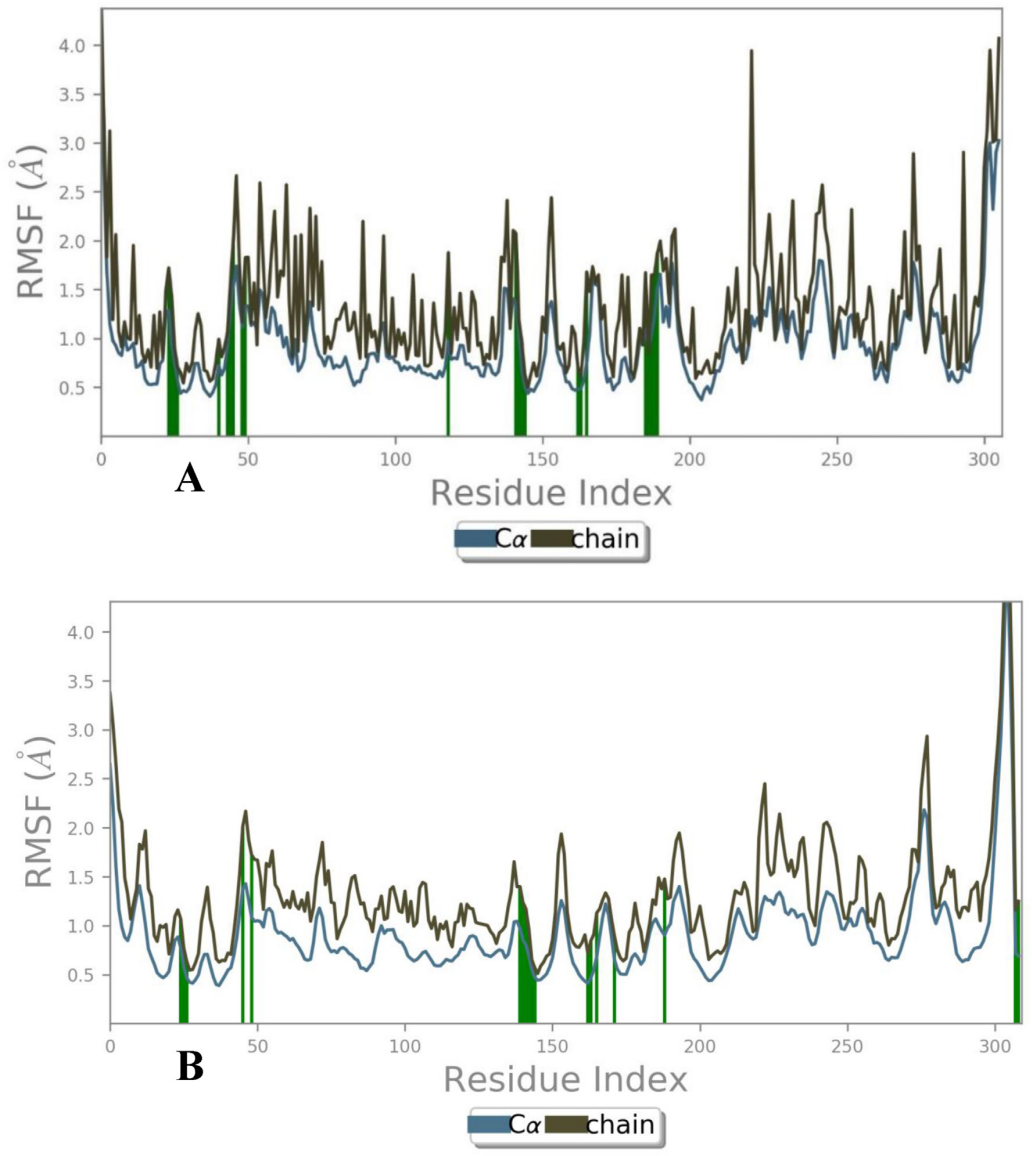
RMSF of M^pro^ (Blue) and sidechain of (brown) are forms H-bond interactions (green line) with Akuammicine N-Oxide. **(A)** Docked copmex **(B)** Native complex.

**Fig. 7:**
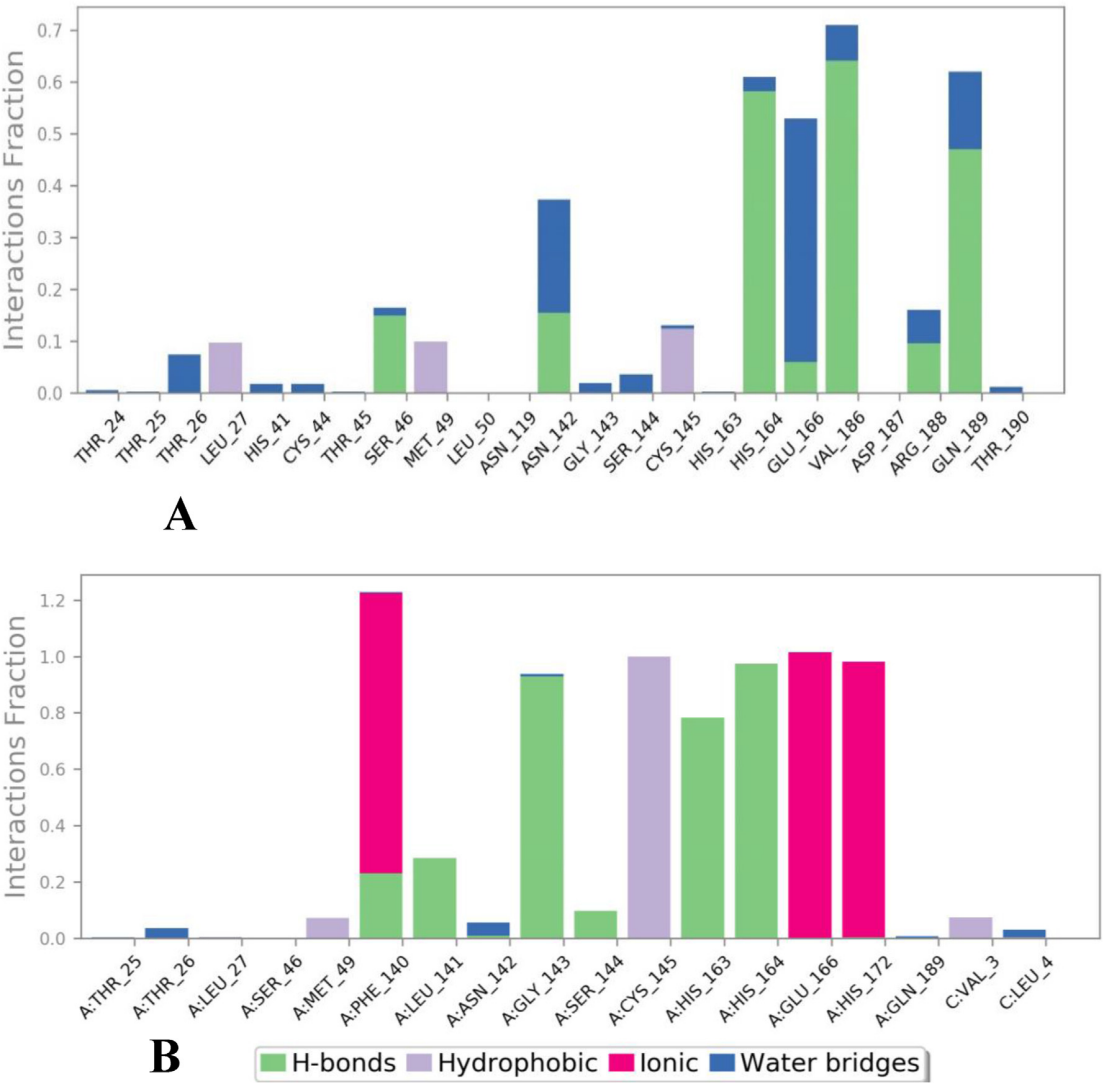
Post molecular dynamics simulations of **(A)** Akuammicine N-Oxideand **(B)** N3 peptide were formed interactions with M^pro^ of SARS-CoV-2.

For understanding the conformational deviations/fluctuations and binding free energy of docked and native complexes, 100 ns period of molecular dynamics simulations was performed for each trajectory. The M^pro^– Akuammicine N-Oxide and native N3 peptide complex depicted the total energy average of −9.2271.69 and −9.5674.02 kcal/mol, potential energy of −114792.08 and −121645.95 kcal/mol, was relatively stable with the average temperature 298.67 K, pressure -0.822 bar, and volume ∼366704.2 Å^3^ throughout the simulations run (Fig. 4). The post MD simulations, each trajectory was analyzed for stability conformation through RMSD, root mean square fluctuation (RMSF), H-bond monitoring and ΔG binding energy (Fig. 5, Fig. 6, Fig. 7 and Supplementary Figs. 1–3).

The average RMSD value observed for M^pro^Cα atoms is 1.4 Ȧ and for proposed Akuammicine N-Oxideis 1.17 Ȧ, which was found stable throughout the simulation period (Fig. 5A). In the native complex of M^pro^ – N3 pepetide, average RMSD values 9.5 Ȧ and 2.05 Ȧ were observed for the Cα atoms and maximum RMSD of M^pro^ Cα atoms is 14.12 Ȧ, that constitutessignificantly not stable during MD simulations (Fig. 5B). To measure the atomic fluctuations in M^pro^ residues, RMSF values were analysed for each residue Cα atoms bound with side-chain of docked and native complex of M^pro^ of SARS-CoV-2. The average RMSF observed for each Cα atoms: 1.4 Ȧ and side chain: 2.5 Ȧ in the overall simulation run docked with Akuammicine N-Oxide (green lines) (Fig. 6A). In the native N3 peptide (green lines) with M^pro^ complex observed in the 1000 frames of MD simulations where the results revealed with average RMSF values for Cα and side-chain have 3.77 Ȧ and 4.52 Ȧ respectively (Fig. 6B).

Notably, post dynamics simulation of the M^pro^ with Akuammicine N-Oxide and native N3 peptide, formed significant H-bonds, hydrophobic and water associated bridges correlating with co–crystal structure (Fig. 7). The Akuammicine N-Oxide formed seven H-bonds in M^pro^ with Ser46 (∼7%), Asn142 (∼7%), His164 (∼27%), Glu166 (∼3%), Val186 (∼30%), Arg188 (∼4%) and Gln189 (∼22%); three hydrophobic interactions with Leu27 (∼30%), Met49 (∼31%) and Cys145 (∼39%); and more than 10% of water associated bridges with Asn142 (∼18%), Glu166 (∼39%) and Gln189 (∼12%) within 4Ȧ region of binding site (Fig. 7A). The co–crystal N3 peptide molecular interaction was substituted with the above furnished residue interactions of Akuammicine N-Oxide with M^pro^ of SARS-COV-2.

While checking the H-bond occupancies over the course of MD simulation trajectories, Phe140 (∼28%), Leu141 (∼28%), Gly143 (∼92%), Ser144 (∼15%), His163 (∼78%), and His164 (∼97%) was observed with native N3 peptide. In another instance, residue Phe140 (∼90%), Glu166 (∼97%) and His172 (∼94%) constructed M^pro^ – N3 peptide contacts via ionic bond occupancies with 1000 frames. Moreover, M^pro^ residues has constituted the hydrophobic: Val3 (∼10%), Met49 (∼10%), Cys145 (∼97%), and water bridges: Leu4 (∼10%) Thr26 (∼10%) with native N3 peptide.

From the comparative MM-GBSA plots between docked M^pro^–Akuammicine N-Oxide and native M^pro^–N3 peptide complexes, in total of 1000 frames obtained from 100 ns MD simulations were considered for binding free energy calculations (ΔG_bind_) (Supplementary Fig. 1). The docked complex illustrated the stable average binding free energy of −44.57 kcal/mol (Supplementary Fig. 1A) than fluctuated native complex with average binding free energy of −38.47 kcal/mol (Supplementary Fig. 1B). The stable ΔG_bind_ values obtained from docked complex is self explanatory that, the proposed M^pro^–Akuammicine N-Oxide exhibited significantly stable and higher binding free energy in comparison with the native complex during 100 ns of MD simulations.

While M^pro^ of secondary structure elements (SSE) contributing to the overall protein stability was examined and noticed that, the protein was maintained an average of ∼42% SSE, mostly composed of ∼18.08% helices and ∼24.14% of strand during 100ns simulation period. Remarkably, the Akuammicine N-Oxide also showed the substantial ligand properties of Radius of Gyration (Rg: ∼4.0), Molecular Surface Area (MolSA: ∼363.37), Solvent Accessible Surface Area (SASA: ∼209.8) and Polar Surface Area (PSA: ∼138.47) (Supplementary Fig. 2). Significantly, one intra-molecular interaction was observed during the simulation time of 100 ns period. Moreover, every rotatable bond of the Akuammicine N-Oxide formed H-bonds with M^pro^ was stable throughout the simulation trajectory (Supplementary Fig. 3). To summarize from all the observed findings of molecular docking and dynamics simulations, M^pro^ – Akuammicine N-Oxide complex was highly stable and shown significant molecular interactions when compared with native M^pro^ – N3 pepetide complex of SARS-COV-2.

## 4 Discussion

M^pro^ is one of the best-characterized pan drug target for the coronavirus's family. Along with M^pro^ an essential enzyme for the endurance of virus, processing the polyproteins which are translated from the viral RNA [[13], [14], [15]]. During translation, the M^pro^ exclusively cleaves at all 11 cleavage sites (after a glutamine residue) of polypeptide sequences on the large polyprotein named as 1 ab (replicase 1-ab, ∼790 kDa); Inhibiting the activity of this enzyme would block the viral replication. The cleavage site recognition sequence at most sites is Leu–Gln (Ser, Ala, Gly). To the best of our knowledge no human proteases with a similar cleavage site specificity are known, such inhibitors are unlikely to be toxic [[13], [14], [15]].

In this present study, we performed different docking strategies of Discovery studio and Auto Dock Vina, M^pro^ of 6LU7 with 36 compounds of AYUSH-64. The results revealed that, 35 compounds have a good binding affinity and strong H-bond than the native N3 peptide bound with the M^pro^ of SASR-CoV-2 binding site (Supplementary Table 1 and Fig. 2). The top scored docking pose compound, Akuammicine N-Oxide formed fourteen H-bond with M^pro^ side chain residues along number bonds mentioned within brackets, His41 (2), Met49 (2), Tyr54, Asn142 (2), Gly143, Ser144, Cys145 (2), His163, Met165, and His172 of M^pro^ significant binding site residues (Fig. 3). The Akuammicine N-Oxide formed vdW interactions with the binding site residues of Cys44, Pro52, Phe140, Leu141, Asn142, Arg188, Asp187, and Gln189 within 4 Å region in both docking strategies (Supplementary Table 2 and Fig. 3B). Thus, the molecular docking approaches utile to identify the novel hits/leads against several pathogenesis. Docking aims to reproduce a conformational selection specific to target conformations that are recognized by the hit/lead to form molecular-interactions in the form of thermodynamical favored complex. The MD simulations were successfully used to achieve the major objective of substantially stabilize the M^pro^ quaternary structure which involves an increase of the inter-molecular interactions by strengthening the ionic contacts and structural flexibility of the M^pro^ binding pocket to allow flexible exploration of its conformational space, thus extracting representative frame from docked complexes.

To investigate the top scored docking pose complex (M^pro^ – Akuammicine N-Oxide) binding conformational stability with SARS-CoV-2 M^pro^, we performed 100 ns MD simulations using Desmond. The MD simulations results revealed, Akuammicine N-Oxide formed seven stable H-bonds with Ser46, Asn142, His164, Glu166, Val186, Arg188 and Gln189; three hydrophobic interactions with Leu27, Met49 and Cys145; and water associated bridges with Asn142, Glu166 and Gln189 within 4Ȧ region of binding site (Fig. 7A). The co–crystal N3 peptide molecular interaction was substituted with Akuammicine N-Oxide in M^pro^ of SARS-COV-2. Furthermore, advanced MD simulations of MM-GBSA analysis (total frames obtained from 100 ns run) also confirmed the proposed M^pro^–Akuammicine N-Oxide complex has consistent binding free energy (ΔG_bind_) score when compared to native complex. This study from sequence levels to advanced structural dynamics confirmed, Akuammicine N-Oxide of AYUSH-64 have potential inhibitor against SARSR-CoV-2. Superimposition of experimentally derived X-ray crystal structure of the M^pro^ of MERS-CoV, SARS-CoV, and SARS-CoV-2, shares structural similarity and high conservativeness of substrate binding site. These observances are enlightened; Akuammicine N-Oxide was a potential pan-coronaviral inhibitor against corona family. Although these results were obtained from *in silico* approaches, the formation of these interactions provides insight to design and repurposing of novel drugs that might enlighten scientific community and researchers for further immune-modulatory investigations in human. Further, it is pertinent to note that AYUSH-64 is a compound herbal preparation, which is already tested to some extent for its safety and efficacy for treating malaria, joint disorders and influenza like illness. The currents *in silico* study provide a valid basis for its repurposing in the management of COVID-19.

## 5 Conclusion

This study revealed that, several compounds from an Ayurvedic drug are acting as potent inhibitors of SARS-CoV-2 M^pro^ enzyme. Assessing the individual compounds through two different molecular docking strategies and extensive molecular dynamics simulations, explains that the Akuammicine N-Oxide is tightly bound (H-bond) with significant residues Cys145 and His164 of M^pro^, also found stable during pre- and post-dynamics simulations. The results indicate that AYUSH-64, an approved and safe drug for managing joint pains, fever, influenza like illnesses make it a good candidate for repurposing against COVID-19. Therefore, AYUSH-64 might be validated through further experimental, clinical studies that entailed to confirm the anti-protease properties against COVID-19.

## Source(s) of Funding

None.

## Conflicts of Interest

None.

## References

[1] Rabi F.A., Al Zoubi M.S., Al-Nasser A.D., Kasasbeh G.A., Salameh D.M. (2020). Sars-cov-2 and coronavirus disease 2019: what we know so far. Pathogens.

[2] Wu F., Zhao S., Yu B., Chen Y.M., Wang W., Song Z.G. (2020). A new coronavirus associated with human respiratory disease in China. Nature.

[3] Zhou P., Lou Yang X., Wang X.G., Hu B., Zhang L., Zhang W. (2020). A pneumonia outbreak associated with a new coronavirus of probable bat origin. Nature.

[4] Lu R., Zhao X., Li J., Niu P., Yang B., Wu H. (2020). Genomic characterisation and epidemiology of 2019 novel coronavirus: implications for virus origins and receptor binding. Lancet.

[5] Anand K., Ziebuhr J., Wadhwani P., Mesters J.R., Hilgenfeld R. (2003). Coronavirus main proteinase (3CLpro) Structure: basis for design of anti-SARS drugs. Science (80- ).

[6] Jin Z., Du X., Xu Y., Deng Y., Liu M., Zhao Y. (2020). Structure of Mpro from SARS-CoV-2 and discovery of its inhibitors. Nature.

[7] Li C.C., Wang X.J., Wang H.C.R. (2019). Repurposing host-based therapeutics to control coronavirus and influenza virus. Drug Discov Today.

[8] Liu A.L., Du G.H. (2012).

[9] Newman D.J., Cragg G.M. (2007). Natural products as sources of new drugs over the last 25 years. J Nat Prod.

[10] Guastalegname M., Vallone A. (2020). Could chloroquine/hydroxychloroquine be harmful in coronavirus disease 2019 (COVID-19) treatment?. Clin Infect Dis.

[11] Enmozhi S.K., Raja K., Sebastine I., Joseph J. (2020). Andrographolide as a potential inhibitor of SARS-CoV-2 main protease: an in silico approach. J Biomol Struct Dyn.

[12] Khaerunnisa S., Kurniawan H., Awaluddin R., Suhartati S. (2020). Potential inhibitor of COVID-19 main protease (M pro) from several medicinal plant compounds by molecular docking study. Preprints.

[13] Khan S.A., Zia K., Ashraf S., Uddin R., Ul-Haq Z. (2020). Identification of chymotrypsin-like protease inhibitors of SARS-CoV-2 via integrated computational approach. J Biomol Struct Dyn.

[14] Khan R.J., Jha R.K., Amera G.M., Jain M., Singh E., Pathak A. (2020). Targeting SARS-CoV-2: a systematic drug repurposing approach to identify promising inhibitors against 3C-like proteinase and 2′-O-ribose methyltransferase. J Biomol Struct Dyn.

[15] Naik V.R., Munikumar M., Ramakrishna U., Srujana M., Goudar G., Naresh P. (2020). Remdesivir (GS-5734) as a therapeutic option of 2019-nCOV main protease - in silico approach. J Biomol Struct Dyn.

[16] Tahir ul Qamar M., Alqahtani S.M., Alamri M.A., Chen L.L. (2020). Structural basis of SARS-CoV-2 3CLpro and anti-COVID-19 drug discovery from medicinal plants. J Pharm Anal.

[17] Bhardwaj V.K., Singh R., Sharma J., Rajendran V., Purohit R., Kumar S. (2020). Identification of bioactive molecules from tea plant as SARS-CoV-2 main protease inhibitors. J Biomol Struct Dyn.

[18] Gurung A.B., Ali M.A., Lee J., Farah M.A., Al-Anazi K.M. (2020). Unravelling lead antiviral phytochemicals for the inhibition of SARS-CoV-2 Mpro enzyme through in silico approach. Life Sci.

[19] CCRAS (1987).

[20] Pandey P.N., Kishore P. (1991). Effect of ayush-64 and saptaparnaghana vati on microfilaraemia. J Res Ayurveda Siddha.

[21] Pandey P.N., Padhi M.M., PK (1991). An epidemiological survey on microfilaraemia in the villages around bhubaneswar with therapeutic effect of ayush-64. J Res Ayurveda Siddha.

[22] Chari M.V., Venkataragbavan S., Sesbadri C., Ramakrisbna B., Sbetty G. (1985). A double blind clinical trial with ayush-64 an ayurvedic drug in p. vivax malaria. J Res Ayurveda Siddha.

[23] Gundeti M.S., Bhurke L.W., Mundada P.S., Murudkar S., Surve A., Sharma R. (2020). AYUSH 64, a polyherbal Ayurvedic formulation in Influenza like Illness: results of a pilot study. J Ayurveda Integr Med.

[24] Cheng T., Li Q., Zhou Z., Wang Y., Bryant S.H. (2012). Structure-based virtual screening for drug discovery: a problem-centric review. AAPS J.

[25] Song Chun Meng, SJL, JC (2009). Recent advances in computer-aided drug design. Briefings Bioinf.

[26] Shukla S., Mehta A., Mehta P., Vyas S.P., Shivaprasad H.N. (2010). In vivo immunomodulatory activities of the aqueous extract of bonduc nut Caesalpinia bonducella seeds. Pharm Biol.

[27] Pradeepkiran J.A., Kumar K.K., Kumar Y.N., Bhaskar M. (2015). Modeling, molecular dynamics, and docking assessment of transcription factor rho: a potential drug target in brucella melitensis 16M. Drug Des Dev Ther.

[28] Pradeepkiran J.A., Yellapu N.K., Matcha B. (2016). Modeling, molecular docking, probing catalytic binding mode of acetyl-CoA malate synthase G in Brucella melitensis 16M. Biochem Biophys Reports.

[29] Diller D.J., Merz K.M. (2001). High throughput docking for library design and library prioritization. Proteins Struct Funct Genet.

[30] Munikumar M., Natarajan P., Amineni U., Radha Krishna K.V. (2019). Discovery of potential lumazine synthase antagonists for pathogens involved in bacterial meningitis: in silico study. Informatics Med Unlocked.

[31] Munikumar M., Krishna V.S., Reddy V.S., Rajeswari B., Sriram D., Rao M.V. (2018). In silico design of small peptides antagonist against leptin receptor for the treatment of obesity and its associated immune-mediated diseases. J Mol Graph Model.

[32] Shan Y., Kim E.T., Eastwood M.P., Dror R.O., Seeliger M.A., Shaw D.E. (2011). How does a drug molecule find its target binding site?. J Am Chem Soc.

[33] Evans D.J., Holian B.L. (1985). The Nose-Hoover thermostat. J Chem Phys.

[34] Martyna G.J., Tobias D.J., Klein M.L. (1994). Constant pressure molecular dynamics algorithms. J Chem Phys.

[35] Lyne P.D., Lamb M.L., Saeh J.C. (2006). Accurate prediction of the relative potencies of members of a series of kinase inhibitors using molecular docking and MM-GBSA scoring. J Med Chem.

[36] Koh Y., Das D., Leschenko S., Nakata H., Ogata-Aoki H., Amano M. (2009). GRL-02031, a novel nonpeptidic protease inhibitor (PI) containing a stereochemically defined fused cyclopentanyltetrahydrofuran potent against multi-PI-resistant human immunodeficiency virus type 1 in vitro. Antimicrob Agents Chemother.

[37] Chakrabarti P., Das B.K., Kapil A. (2009). Application of 16S rDNA based seminested PCR for diagnosis of acute bacterial meningitis. Indian J Med Res.

[38] Koh Y., Das D., Leschenko S., Nakata H., Ogata-Aoki H., Amano M. (2009). GRL-02031, a novel nonpeptidic protease inhibitor (PI) containing a stereochemically defined fused cyclopentanyltetrahydrofuran potent against multi-PI-resistant human immunodeficiency virus type 1 in vitro. Antimicrob Agents Chemother.

[39] Chakrabarti P., Das B.K., Kapil A. (2009). Application of 16S rDNA based seminested PCR for diagnosis of acute bacterial meningitis. Indian J Med Res.

[40] Ramaprasad V., Ramaprasad V. (1998).

[41] Sharma P.V.S.G. (1998).

[42] Sharma P.V.S.G. (1979).

[43] Tripathi Indradeo, Indradeo T. (1998).

[44] Khyade M.S., Kasote D.M., Vaikos N.P. (2014). Alstonia scholaris (L.) R. Br. and Alstonia macrophylla Wall. ex G. Don: a comparative review on traditional uses, phytochemistry and pharmacology. J Ethnopharmacol.

[45] Baliga M.S. (2012). Review of the phytochemical, pharmacological and toxicological properties of Alstonia Scholaris Linn. R. Br (Saptaparna). Chin J Integr Med.

[46] Singh S., Sharma P.K., Kumar N., Dudhe R. (2010). Evaluation of acetone extract of three Indian medicinal plants for schizonticidal properties in Plasmodium falciparum. Int J Pharmacy&Technology IJPT.

[47] Zhang L., Zhang C.J., Zhang D.B., Wen J., Zhao X.W., Li Y. (2014). An unusual indole alkaloid with anti-adenovirus and anti-HSV activities from Alstonia scholaris. Tetrahedron Lett.

[48] Mistry D., Pithawala M. (2018). Protective effect of Alstonia scholaris Linn. R. Br. against Bleomycin induced chromosomal damage in cultured human lymphocytes, in vitro. Drug Chem Toxicol.

[49] Shang J.H., Cai X.H., Feng T., Zhao Y.L., Wang J.K., Zhang L.Y. (2010). Pharmacological evaluation of Alstonia scholaris: anti-inflammatory and analgesic effects. J Ethnopharmacol.

[50] Christina C., Prasanna Kumar S., Margret Beula J., Chandra Lekha N., Jeyaraj N., Ravikumar S. (2015). In vitro antiplasmodial activity of Kani herb Alstonia scholaris against Plasmodium falciparum. Innovat J Med Health Sci.

[51] Gandhi M., Vinayak V.K. (1990). Preliminary evaluation of extracts of Alstonia scholaris bark for in vivo antimalarial activity in mice. J Ethnopharmacol.

[52] Pandey B.L., Das P.K. (1988). Immunopharmacological studies on picrorhiza kurroa royle-ex-benth part III: adrenergic mechanisms of anti-inflammatory action. Indian J Physiol Pharmacol.

[53] Hussain A., Shadma W., Maksood A., Ansari S.H. (2013). Protective effects of Picrorhiza kurroa on cyclophosphamide-induced immunosuppression in mice. Pharmacogn Res.

[54] Sharma M.L., Rao C.S., Duda P.L. (1994). Immunostimulatory activity of Picrorhiza kurroa leaf extract. J Ethnopharmacol.

[55] Gupta A., Khajuria A., Singh J., Bedi K.L., Satti N.K., Dutt P. (2006). Immunomodulatory activity of biopolymeric fraction RLJ-NE-205 from Picrorhiza kurroa. Int Immunopharm.

[56] Kant K., Walia M., Agnihotri V.K., Pathania V., Singh B. (2013). Evaluation of antioxidant activity of Picrorhiza kurroa (leaves) extracts. Indian J Pharmaceut Sci.

[57] Banyal H.S.,.R., Devi N., Banyal Rani H.S., Devi N. (2014). Picrorhiza kurrooa royal ex benth exhibits antimalarial activity against plasmodium berghei vincke and lips, 1948. Asian J Bio Sci.

[58] Lusakibanza M., Mesia G., Tona G., Karemere S., Lukuka A., Tits M. (2010). In vitro and in vivo antimalarial and cytotoxic activity of five plants used in congolese traditional medicine. J Ethnopharmacol.

[59] Nondo R.S.O., Moshi M.J., Erasto P., Masimba P.J., Machumi F., Kidukuli A.W. (2017). Anti-plasmodial activity of Norcaesalpin D and extracts of four medicinal plants used traditionally for treatment of malaria. BMC Compl Alternative Med.

[60] Woo S.Y., Win N.N., Noe Oo W.M., Ngwe H., Ito T., Abe I. (2019). Viral protein R inhibitors from Swertia chirata of Myanmar. J Biosci Bioeng.

[61] Verma H., Patil P., Kolhapure R., Gopalkrishna V. (2008). Antiviral activity of the Indian medicinal plant extract, Swertia chirata against herpes simplex viruses: a study by in-vitro and molecular approach. Indian J Med Microbiol.

[62] Hu T.Y., Ju J.M., Mo L.H., Ma L., Hu W.H., You R.R. (2019). Anti-inflammation action of xanthones from Swertia chirayita by regulating COX-2/NF-κB/MAPKs/Akt signaling pathways in RAW 264.7 macrophage cells. Phytomedicine.

[63] Lad H., Bhatnagar D. (2016). Amelioration of oxidative and inflammatory changes by Swertia chirayita leaves in experimental arthritis. Inflammopharmacology.

[64] Kalauni S.K., Awale S., Tezuka Y., Banskota A.H., Linn T.Z., Asih P.B.S. (2006). Antimalarial activity of cassane- and norcassane-type diterpenes from Caesalpinia crista and their structure–activity relationship. Biol Pharm Bull.

[65] Sarkar R., Hazra B., Mandal N. (2012). Hepatoprotective potential of Caesalpinia crista against iron-overload-induced liver toxicity in mice. Evidence-Based Complement Altern Med.

[66] Kumar Rs, Narasingappa R., Joshi C., Girish T., Danagoudar A. (2017). Caesalpinia Crista Linn. Induces protection against DNA and membrane damage. Phcog Mag.

[67] Arif T., Mandal T.K., Kumar N., Bhosale J.D., Hole A., Sharma G.L. (2009). In vitro and in vivo antimicrobial activities of seeds of Caesalpinia bonduc (Lin.) Roxb. J Ethnopharmacol.

[68] Dhar M.L., Dhar M.M., Dhawan B.N., Mehrotra BN R.C. (1968). Screening of Indian plants for biological activity: I. Indian J Exp Biol.

[69] Sharma K.D., Kapoor M.L., Vaidya Miss S.P., LKS (1980). A clinical trial of Ayush 64 (a coded antimalarial medicine) in cases of malaria. J Res Ayurveda Siddha.

[70] Pandey P.N., Padhi M.M., PK (1991). An epidemiological survey on microfilaraemia in the villages around bhubaneswar with therapeutic effect of ayush-64. J Res Ayurveda Siddha.

[71] Gundeti M.S., Bhurke L.W., Mundada P.S., Murudkar S., Surve A., Sharma R. (2020). AYUSH 64, a polyherbal Ayurvedic formulation in Influenza like Illness: results of a pilot study. J Ayurveda Integr Med.

[72] Sastry J.L.N. (1999). An indigenous herbal formulation to combat Falciparum Malaria. J Indian Syst Med Homeopath.

