# Supplementary material for: In silico evaluation of the compounds of the ayurvedic drug, AYUSH-64, for the action against the SARS-CoV-2 main protease
Source: J Ayurveda Integr Med. 2021 Feb 25;13(1):100413. doi: 10.1016/j.jaim.2021.02.004 (PMC7906523; doi:10.1016/j.jaim.2021.02.004)
Supplement: Multimedia component 1 [file mmc1.docx]

**Supplementary Tables**

**Supplementary Table 1.** Molecular docking score for AYUSH-64 compounds with M^pro^ of 2019-nCoV.

| **Name of the plant** | **Chemical compound name** | **Chemical compound structure** | **LibDock score (kcal/mol)** | **Binding affinity (kcal/mol)** |
| --- | --- | --- | --- | --- |
| *Alstonia scholaris* | Akuammicine N-Oxide | 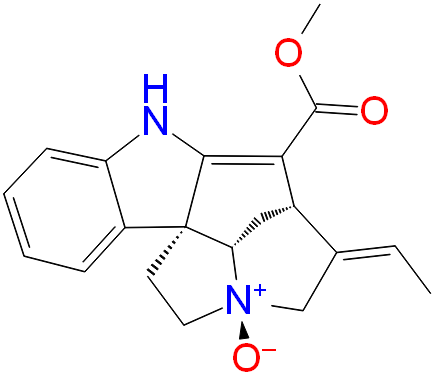 | 147.92 | -8.4 |
| *Alstonia scholaris* | Akuammiginone | 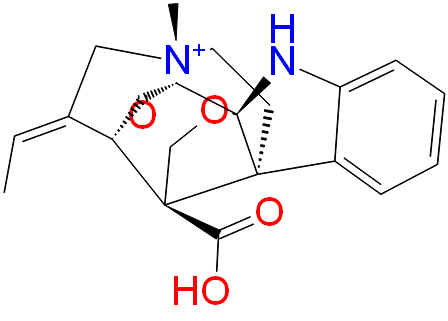 | 146.865 | -7.5 |
| *Alstonia scholaris* | Echitaminic acid | 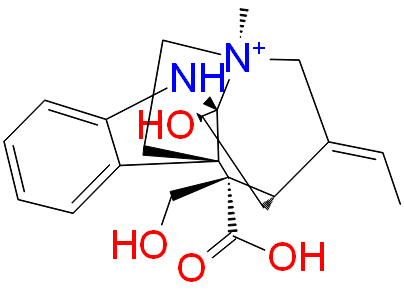 | 146.879 | -7.4 |
| *Alstonia scholaris* | Echitamidine N-oxide | 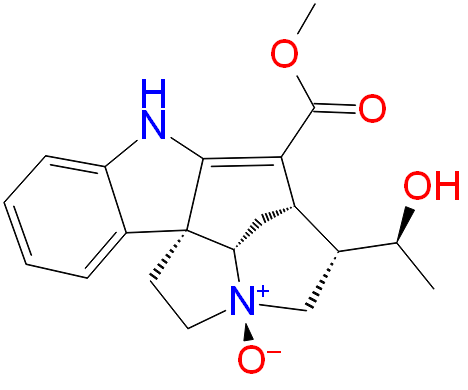 | 145.876 | -7.2 |
| Caesalpina bonducella | Caesalmin G | 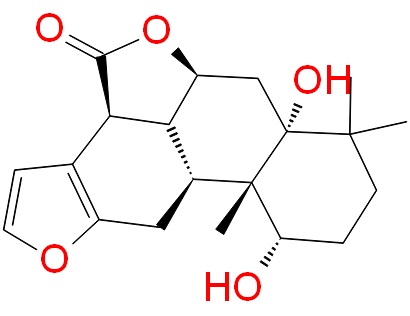 | 144.893 | -7.2 |
| Caesalpina bonducella | Caesalpinin C | 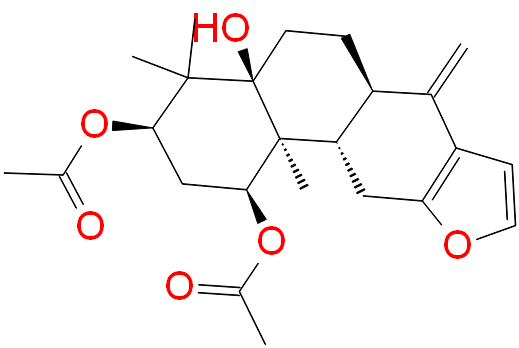 | 144.818 | -7.2 |
| Caesalpina bonducella | [Caesalpinin D](https://pubchem.ncbi.nlm.nih.gov/substance/103636568) | 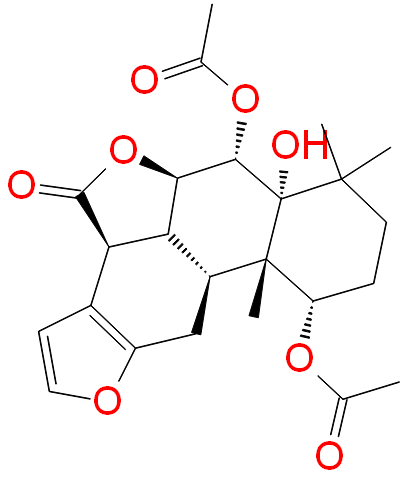 | 143.729 | -7.1 |
| Caesalpina bonducella | [Caesalpinin E](https://pubchem.ncbi.nlm.nih.gov/substance/103636569) | 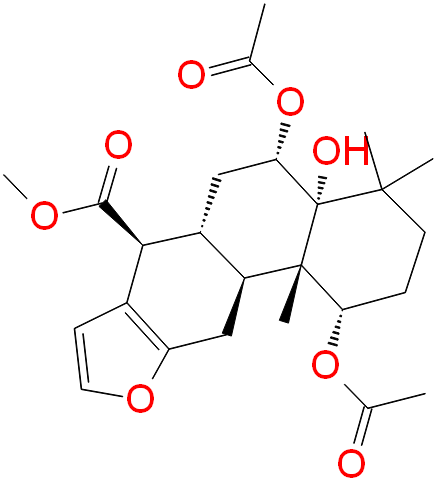 | 143.592 | -7.1 |
| Caesalpina bonducella | [Caesalpinin F](https://pubchem.ncbi.nlm.nih.gov/substance/103636570) | 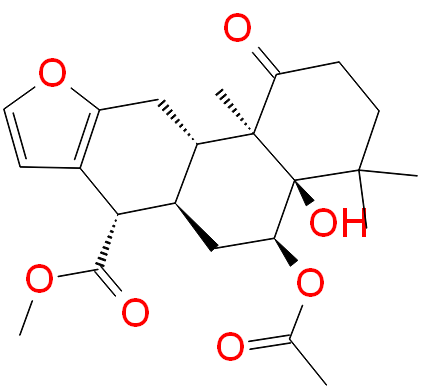 | 142.741 | -7.0 |
| Caesalpina bonducella | [3-O-Acetylnorcaesalpinin A](https://pubchem.ncbi.nlm.nih.gov/substance/103636594) | 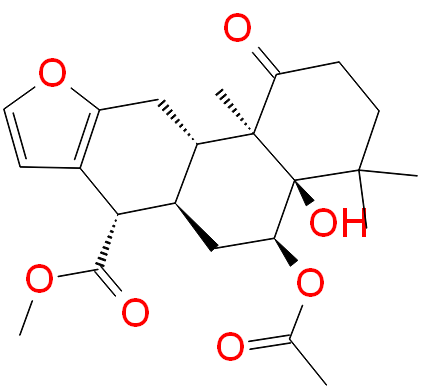 | 142.032 | -7.0 |
| Caesalpina bonducella | [17-Norbonducellpin C](https://pubchem.ncbi.nlm.nih.gov/substance/103636595) | 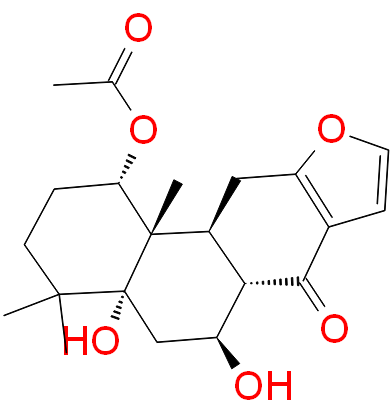 | 140.904 | -6.9 |
| Caesalpina bonducella | [Norcaesalpinin A](https://pubchem.ncbi.nlm.nih.gov/substance/103636596) | 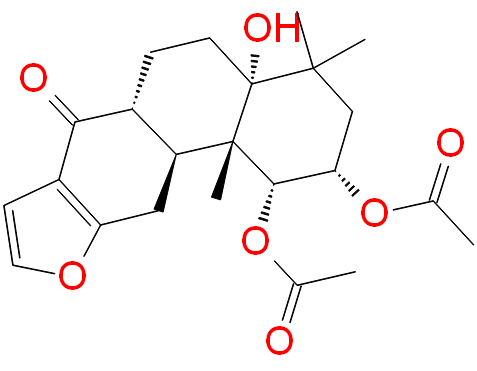 | 142.663 | -6.9 |
| Caesalpina bonducella | [Norcaesalpinin B](https://pubchem.ncbi.nlm.nih.gov/substance/103636597) | 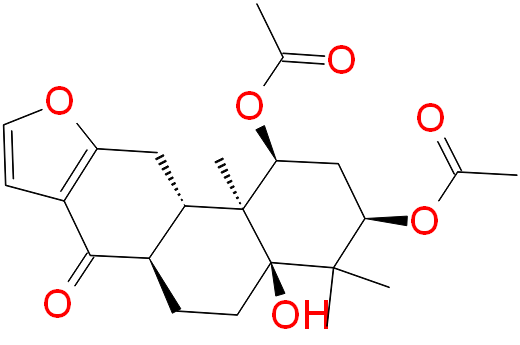 | 139.364 | -6.9 |
| Caesalpina bonducella | [Norcaesalpinin C](https://pubchem.ncbi.nlm.nih.gov/substance/103636610) | 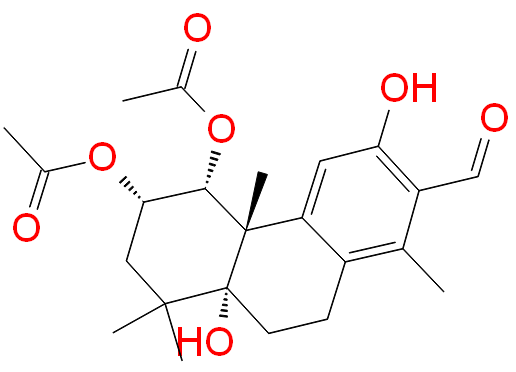 | 137.875 | -6.9 |
| Caesalpina bonducella | 2-Acetoxy-3-deacetoxycaesaldekarin | 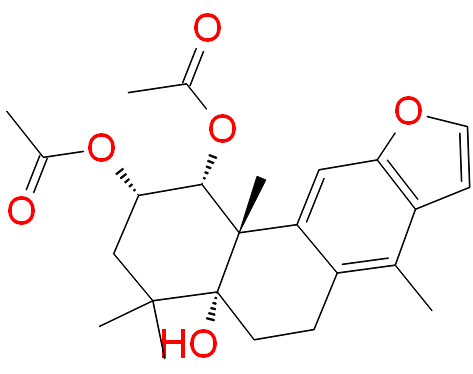 | 138.461 | -6.8 |
| Caesalpina bonducella | [Caesalmin B](https://pubchem.ncbi.nlm.nih.gov/substance/103636612) | 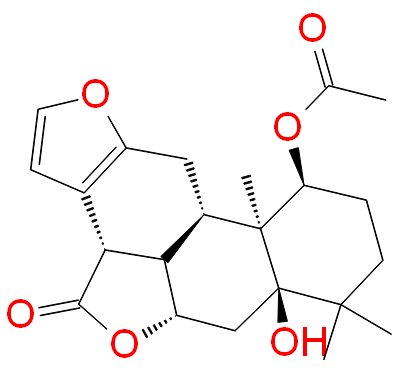 | 134.280 | -6.8 |
| Caesalpina bonducella | [Caesaldekarine](https://pubchem.ncbi.nlm.nih.gov/substance/103636613) | 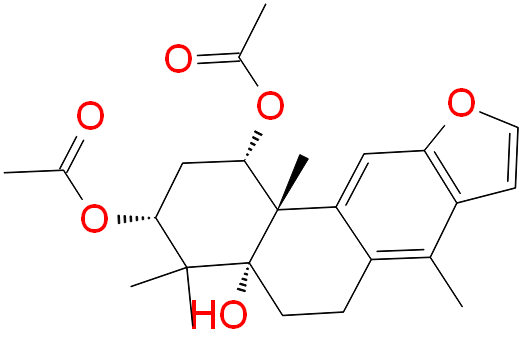 | 134.092 | -6.8 |
| Caesalpina bonducella | [14(17)-Dehydrocaesalpin F](https://pubchem.ncbi.nlm.nih.gov/substance/103636629) | 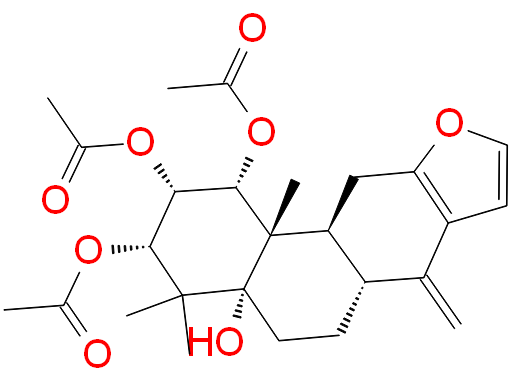 | 132.851 | -6.8 |
| Caesalpina bonducella | 2-Acetoxycaesaldekarine | 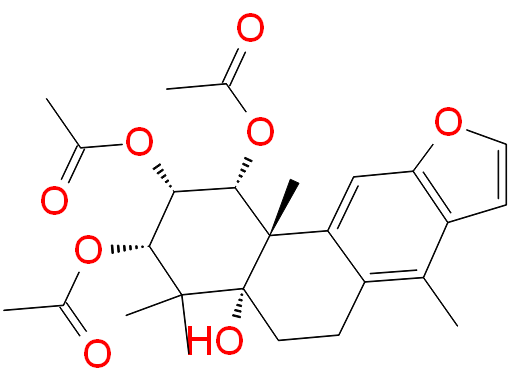 | 131.894 | -6.7 |
| Caesalpina bonducella | Acetoxybonducellpin C | 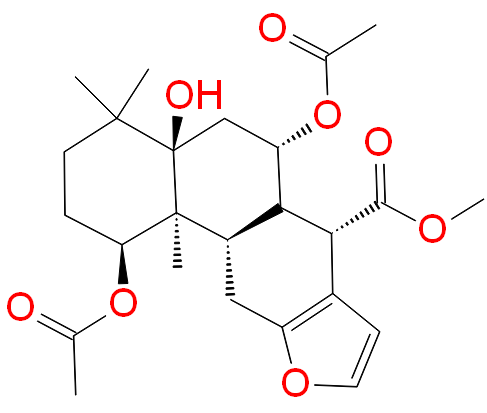 | 135.023 | -6.7 |
| *Swertia chirata* | [Oleanolic acid](https://pubchem.ncbi.nlm.nih.gov/compound/10494) | 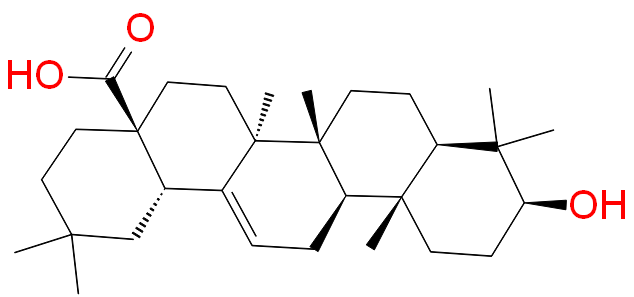 | 130.999 | -6.7 |
| *Swertia chirata* | Amarogentin | 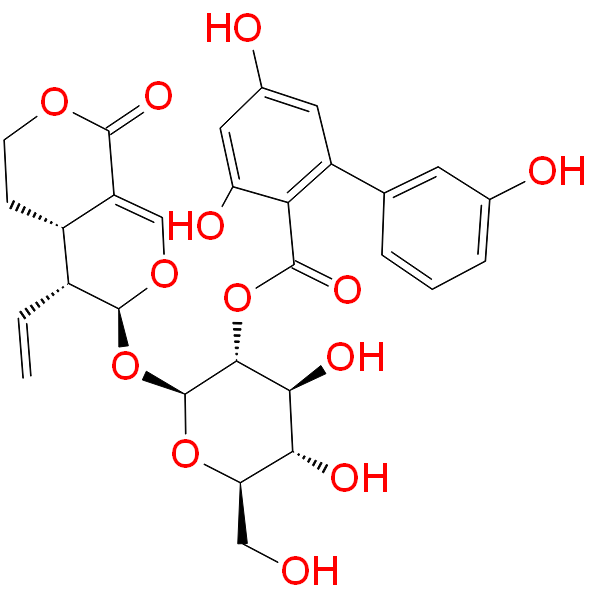 | 130.871 | -6.6 |
| *Swertia chirata* | [Sweroside](https://pubchem.ncbi.nlm.nih.gov/compound/161036) | 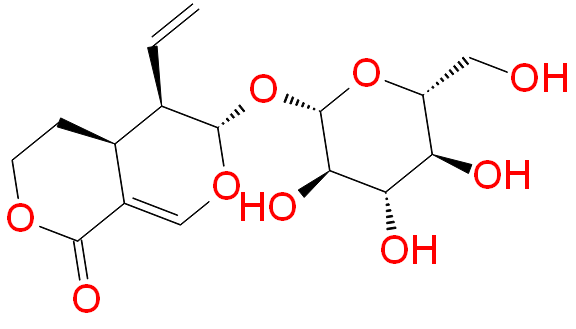 | 130.653 | -6.6 |
| *Picrirrorrhiza kurroa* | [Kutkoside](https://pubchem.ncbi.nlm.nih.gov/compound/182265) | 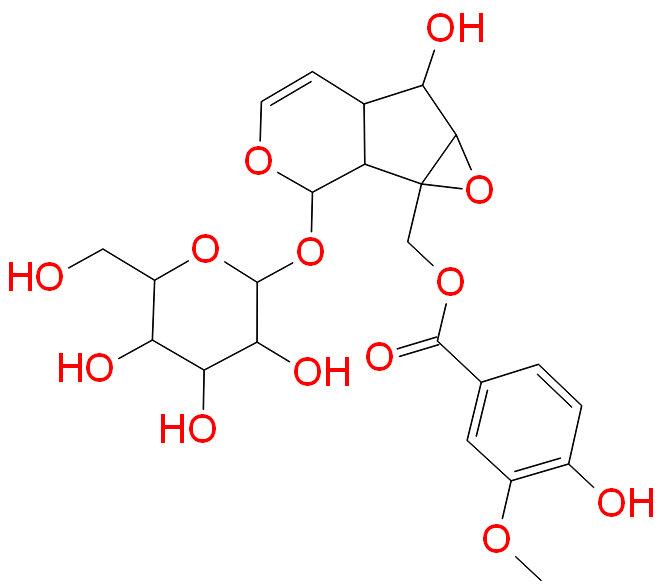 | 129.660 | -6.5 |
| *Picrirrorrhiza kurroa* | [Pikuroside](https://pubchem.ncbi.nlm.nih.gov/compound/21588222) | 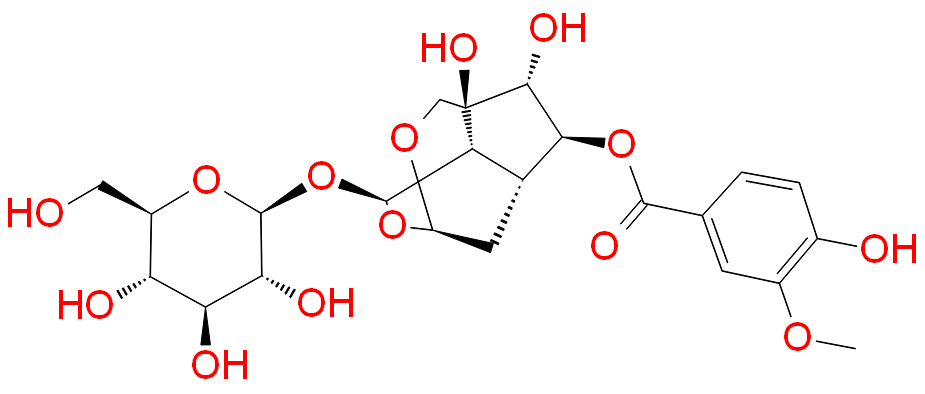 | 129.242 | -6.4 |
| *Alstonia scholaris* | [N(4)-Demethylalstogustine](https://pubchem.ncbi.nlm.nih.gov/compound/21589744) | 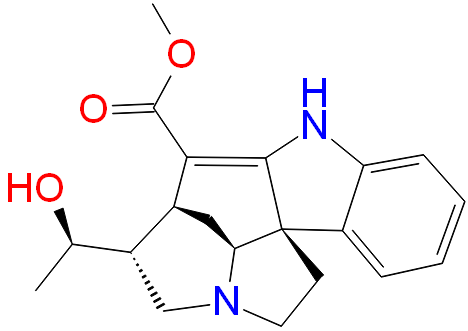 | 128.905 | -6.4 |
| *Picrirrorrhiza kurroa* | [Acetovanillone; Apocynin](https://pubchem.ncbi.nlm.nih.gov/compound/2214) | 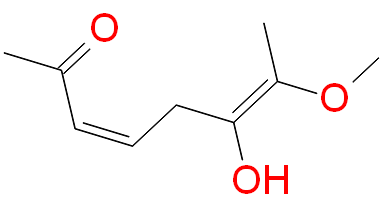 | 130.986 | -6.4 |
| *Picrirrorrhiza kurroa* | [Picroside IV](https://pubchem.ncbi.nlm.nih.gov/compound/23928135) | 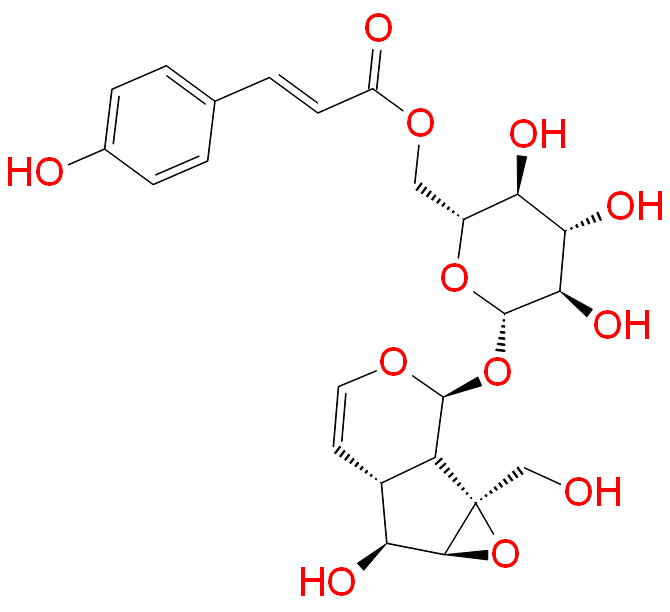 | 130.284 | -6.3 |
| *Picrirrorrhiza kurroa* | [Picroside II](https://pubchem.ncbi.nlm.nih.gov/compound/3081484) | 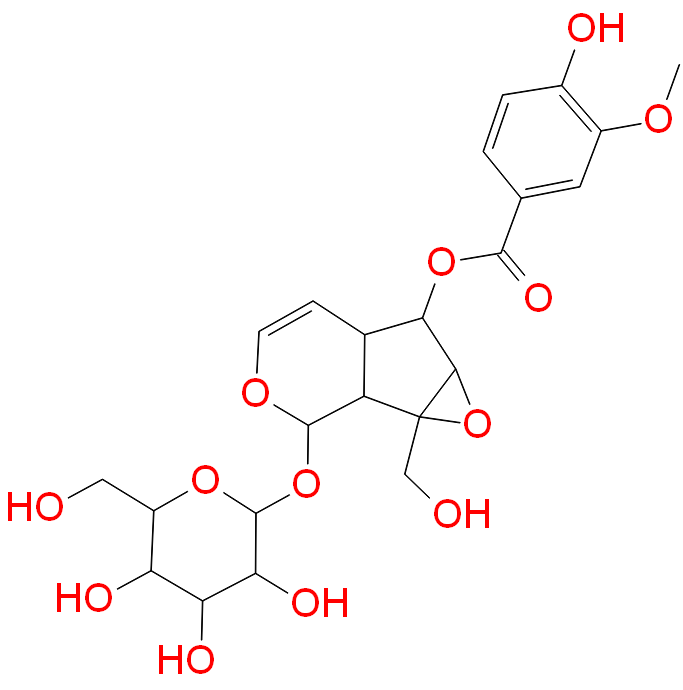 | 129.571 | -6.2 |
| *Swertia chirata* | [Swerchirin; Methylbellidifolin](https://pubchem.ncbi.nlm.nih.gov/compound/5281660) | 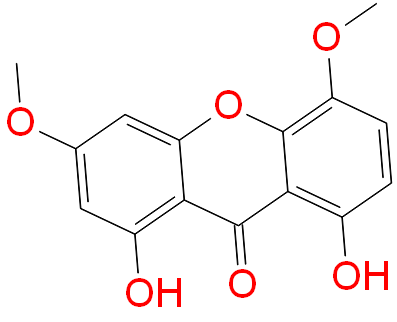 | 129.242 | -6.1 |
| *Alstonia scholaris* | Echitamine | 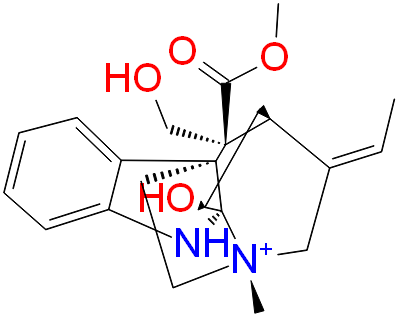 | 128.542 | -6.1 |
| *Picrirrorrhiza kurroa* | [Picroside I](https://pubchem.ncbi.nlm.nih.gov/compound/6440892) | 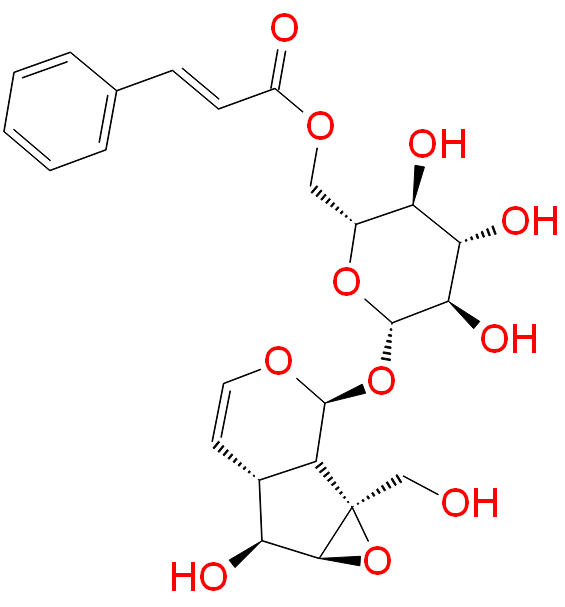 | 127.574 | -5.8 |
| *Swertia chirata* | Ursolic Acid | 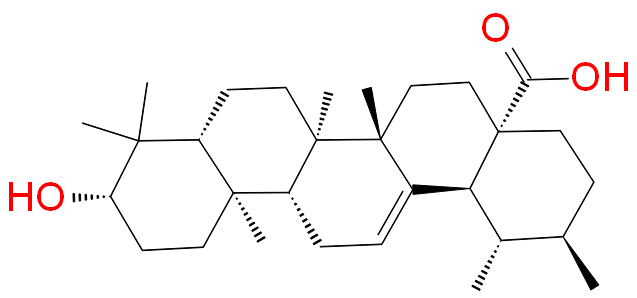 | 112.177 | -5.8 |
| *Picrirrorrhiza kurroa* | Vanillic Acid | 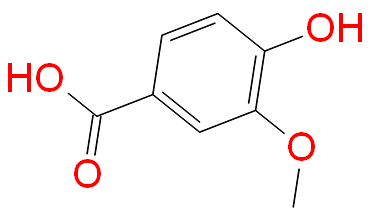 | 111.761 | -5.7 |
| *Picrirrorrhiza kurroa* | Gentiopicroside; Gentiopicrin | 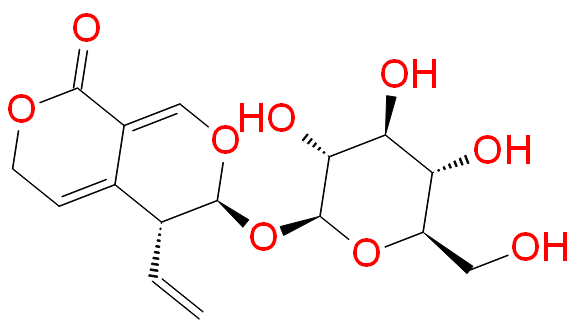 | 111.231 | -4.9 |
| *N3 peptide* | N-[(5-methylisoxazol-3-yl)carbonyl]alanyl-l-valyl-n~1~-((1r,2z)-4-(benzyloxy)-4-oxo-1-{[(3r)-2-oxopyrrolidin-3-yl]methyl}but-2-enyl)-l-leucinamide | 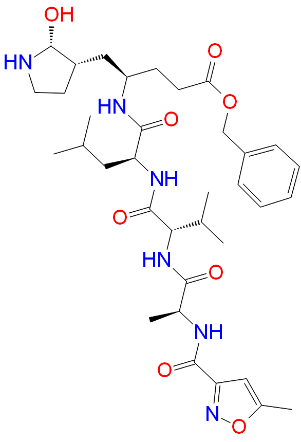 | 105.980 | -4.1 |
| *Alstonia scholaris* | Nb-Demethylalstogustine_N-oxide | 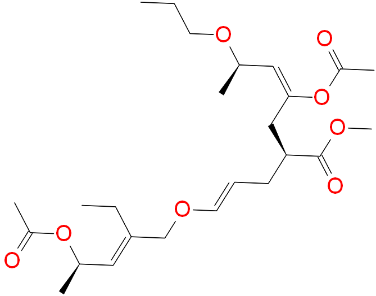 | 74.525 | -3.5 |

**Supplementary Table 2:** The absorption, distribution, metabolism, excretion, and toxicity (ADME/T) predictions for 36 selected AYUSH-64 compounds.

| **Compound name** | **Solubility level ^a^** | **BBB ^b^** | **Hepatotoxicity ^c^** | **Absorption level ^d^** | **CYP2D6 ^e^** | **PPB level ^f^** | **AlogP98** | **PSA_2D** |
| --- | --- | --- | --- | --- | --- | --- | --- | --- |
| Akuammicine N-Oxide | 3 | 3 | 0 | 0 | 0 | 0 | 3.004 | 86.396 |
| akuammiginone | 5 | 3 | 0 | 0 | 0 | 0 | -0.636 | 94.401 |
| echitaminic acid | 3 | 3 | 0 | 0 | 0 | 0 | 1.164 | 47.193 |
| Echitamidine N-oxide | 4 | 3 | 0 | 0 | 0 | 0 | 0.645 | 101.796 |
| Caesalmin G | 4 | 3 | 0 | 0 | 0 | 0 | 1.888 | 80.306 |
| Caesalpinin C | 3 | 2 | 0 | 0 | 0 | 1 | 3.046 | 82.207 |
| Caesalpinin D | 3 | 4 | 0 | 0 | 0 | 0 | 3.81 | 96.552 |
| Caesalpinin E | 2 | 1 | 0 | 0 | 0 | 0 | 4.331 | 70.321 |
| Caesalpinin F | 2 | 2 | 0 | 0 | 0 | 0 | 3.343 | 61.391 |
| 3-O-Acetylnorcaesalpinin A | 4 | 4 | 0 | 0 | 0 | 1 | 1.81 | 120.323 |
| 17-Norbonducellpin C | 3 | 3 | 0 | 0 | 0 | 1 | 2.117 | 82.207 |
| Norcaesalpinin A | 3 | 3 | 0 | 0 | 0 | 1 | 2.451 | 91.137 |
| Norcaesalpinin B | 4 | 3 | 0 | 0 | 0 | 0 | 1.99 | 91.137 |
| Norcaesalpinin C | 3 | 3 | 0 | 0 | 0 | 1 | 2.942 | 91.137 |
| 2-Acetoxy-3-deacetoxycaesaldekarin E | 3 | 2 | 0 | 0 | 0 | 1 | 3.958 | 70.321 |
| Caesalmin B | 3 | 2 | 0 | 0 | 0 | 1 | 3.7 | 70.321 |
| Caesaldekarine | 3 | 2 | 0 | 0 | 0 | 0 | 3.93 | 70.321 |
| 14(17)-Dehydrocaesalpin F | 4 | 3 | 0 | 0 | 0 | 0 | 1.961 | 108.438 |
| 2-Acetoxycaesaldekarine | 4 | 3 | 0 | 0 | 0 | 0 | 2.909 | 96.552 |
| Acetoxybonducellpin C | 3 | 3 | 0 | 0 | 0 | 0 | 3.462 | 96.552 |
| Oleanolic acid | 1 | 4 | 0 | 3 | 0 | 1 | 7.422 | 41.631 |
| Amarogentin | 4 | 4 | 0 | 3 | 0 | 0 | -2.739 | 148.168 |
| Sweroside | 5 | 4 | 0 | 3 | 0 | 0 | -1.698 | 160.054 |
| Kutkoside | 4 | 4 | 0 | 3 | 0 | 0 | -2.881 | 186.844 |
| Pikuroside | 4 | 4 | 0 | 3 | 0 | 0 | -4.979 | 223.059 |
| N(4)-Demethylalstogustine | 3 | 3 | 0 | 0 | 0 | 0 | 0.63 | 86.396 |
| Acetovanillone; Apocynin | 4 | 3 | 0 | 0 | 0 | 0 | 0.732 | 47.046 |
| Picroside IV | 3 | 4 | 0 | 1 | 0 | 0 | 0.073 | 127.912 |
| Picroside II | 4 | 4 | 0 | 3 | 0 | 0 | -3.271 | 198.729 |
| Swerchirin; Methylbellidifolin | 4 | 3 | 0 | 0 | 0 | 0 | 1.01 | 64.906 |
| Echitamine | 3 | 2 | 0 | 0 | 0 | 0 | 1.622 | 47.193 |
| Picroside I | 2 | 3 | 0 | 0 | 0 | 0 | 1.19 | 107.097 |
| Ursolic Acid | 1 | 4 | 0 | 3 | 0 | 1 | 7.866 | 41.631 |
| Vanillic Acid | 4 | 3 | 0 | 0 | 0 | 0 | 0.564 | 67.861 |
| Gentiopicroside; Gentiopicrin | 4 | 4 | 0 | 0 | 0 | 0 | -0.884 | 109.492 |
| Nb-Demethylalstogustine_N-oxide | 4 | 3 | 0 | 0 | 0 | 0 | -0.185 | 59.491 |

^a^0, 1, 2, 3, 4, and 5 denote extremely low, very low but possible, low, good, optimal, and too soluble, respectively. ^b^0, 1, 2, 3, and 4 denote very high, high, medium, low, and undefined, respectively.

^c^0 and 1 represent nontoxic and toxic, respectively. ^d^0, 1, 2, and 3 denote good absorption, moderate absorption, low absorption, and very low absorption, respectively. ^e^0 and 1 denote noninhibitor and inhibitor, respectively. ^f^0, 1, and 2 indicate <90% binding, ≥90% binding, and ≥95% binding, respectively.

**Supplementary Table 3:** Molecular docking interactions of Akuammicine N-Oxide with M^pro^ of SARS-CoV-2.

| **Compound name** | **H-Bonds** | **Bond length (Ȧ)** | **VdW interactions** |
| --- | --- | --- | --- |
| Akuammicine N-Oxide | His41 🡨 C | 4.21 | Cys44, Pro52, Phe140, Leu141, Asn142, Arg188, Asp187, Gln189. |
|  | His41 🡨 C | 3.51 |  |
|  | Met49:O 🡨 H29 | 2.36 |  |
|  | Met49 🡨 C6 | 3.28 |  |
|  | Tyr54: OH 🡨 H46 | 2.47 |  |
|  | Asn142:HD21 🡪 O24 | 1.88 |  |
|  | Asn142: HA 🡪 O23 | 2.12 |  |
|  | Gly143:HN🡪 O23 | 2.41 |  |
|  | Ser144:HN 🡪 O1 | 2.72 |  |
|  | Cys145:HN 🡪 O1 | 2.12 |  |
|  | Cys145 🡪 OH | 3.1 |  |
|  | His163 🡪 C21 | 3.1 |  |
|  | Met165 🡨 C | 3.52 |  |
|  | His172 🡪 C21 | 3.59 |  |
|  |  |  |  |

**Supplementary Figures**


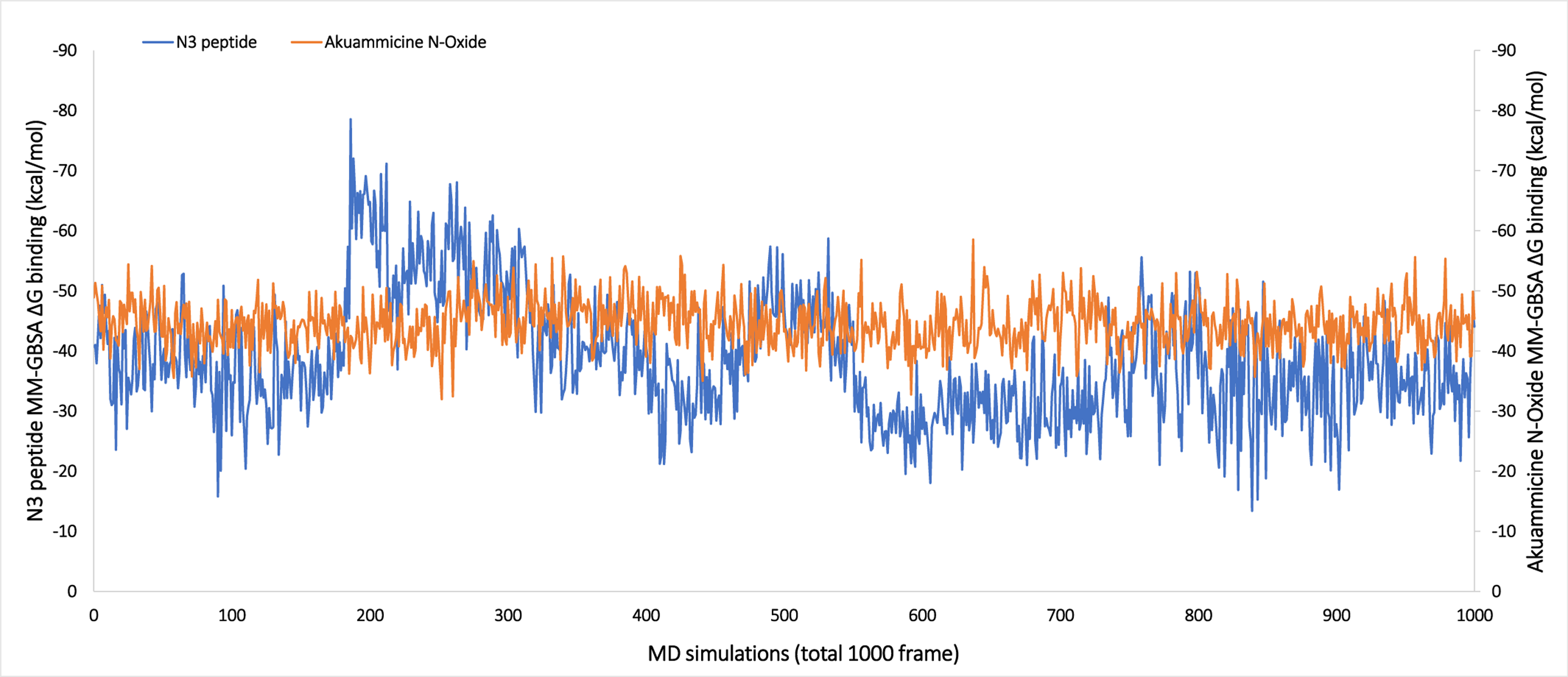


**Supplementary Figure 1.** Comparative MM-GBSA plot for Akuammicine N-Oxide (orange) and native N3 peptide (blue) with M^pro^ of SARS-CoV-2 against the 1000 frames of the 100 ns trajectory.


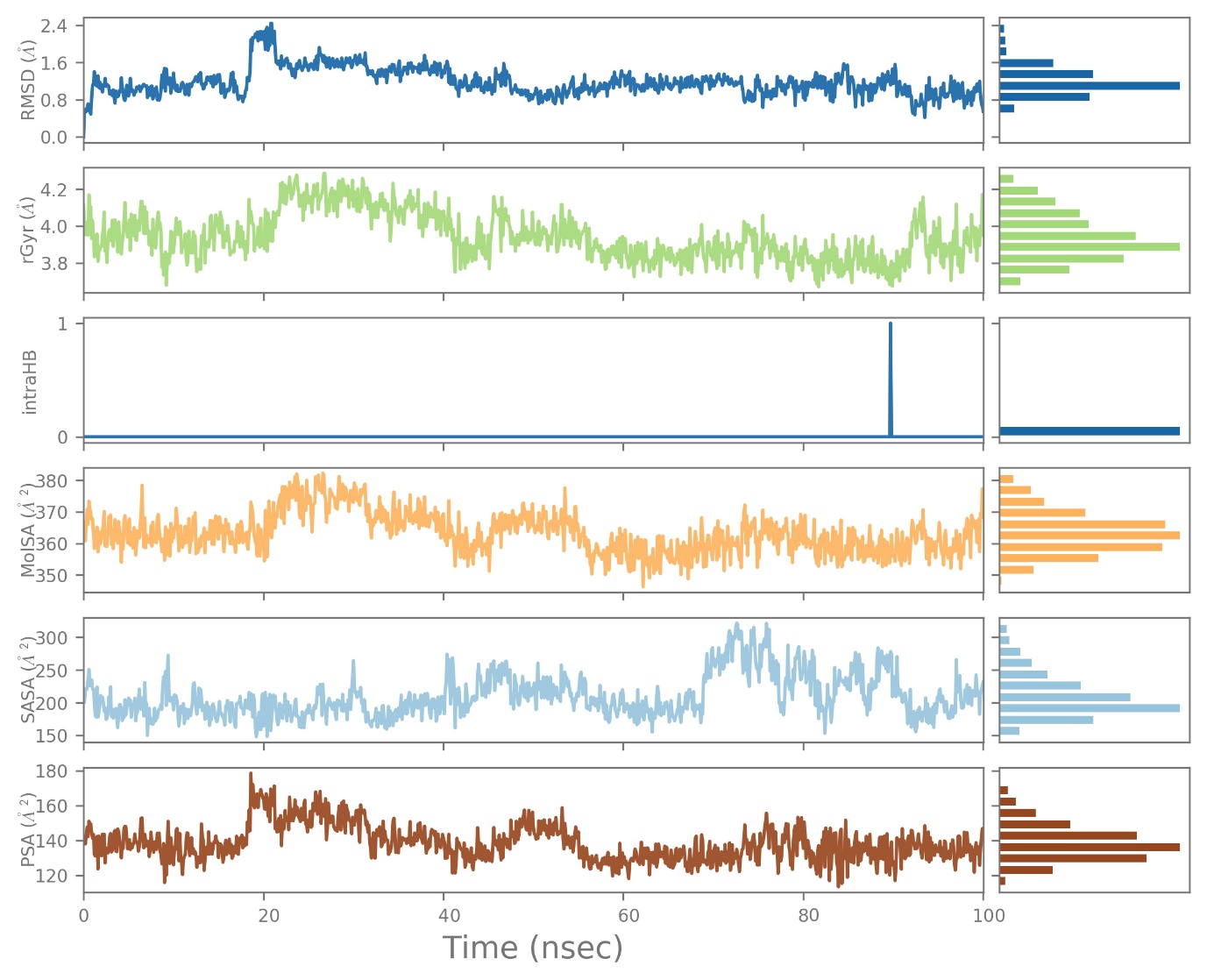


**Supplementary Figure 2**: Ligand properties of M^pro^ with Akuammicine N-Oxide over 100ns of simulation time period.


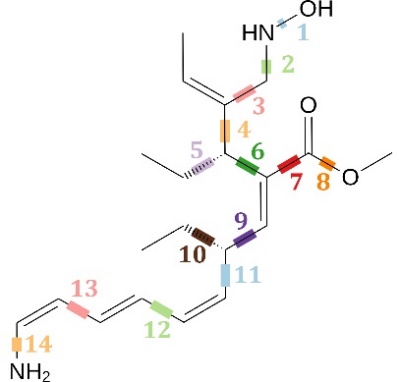


Akuammicine N-Oxide


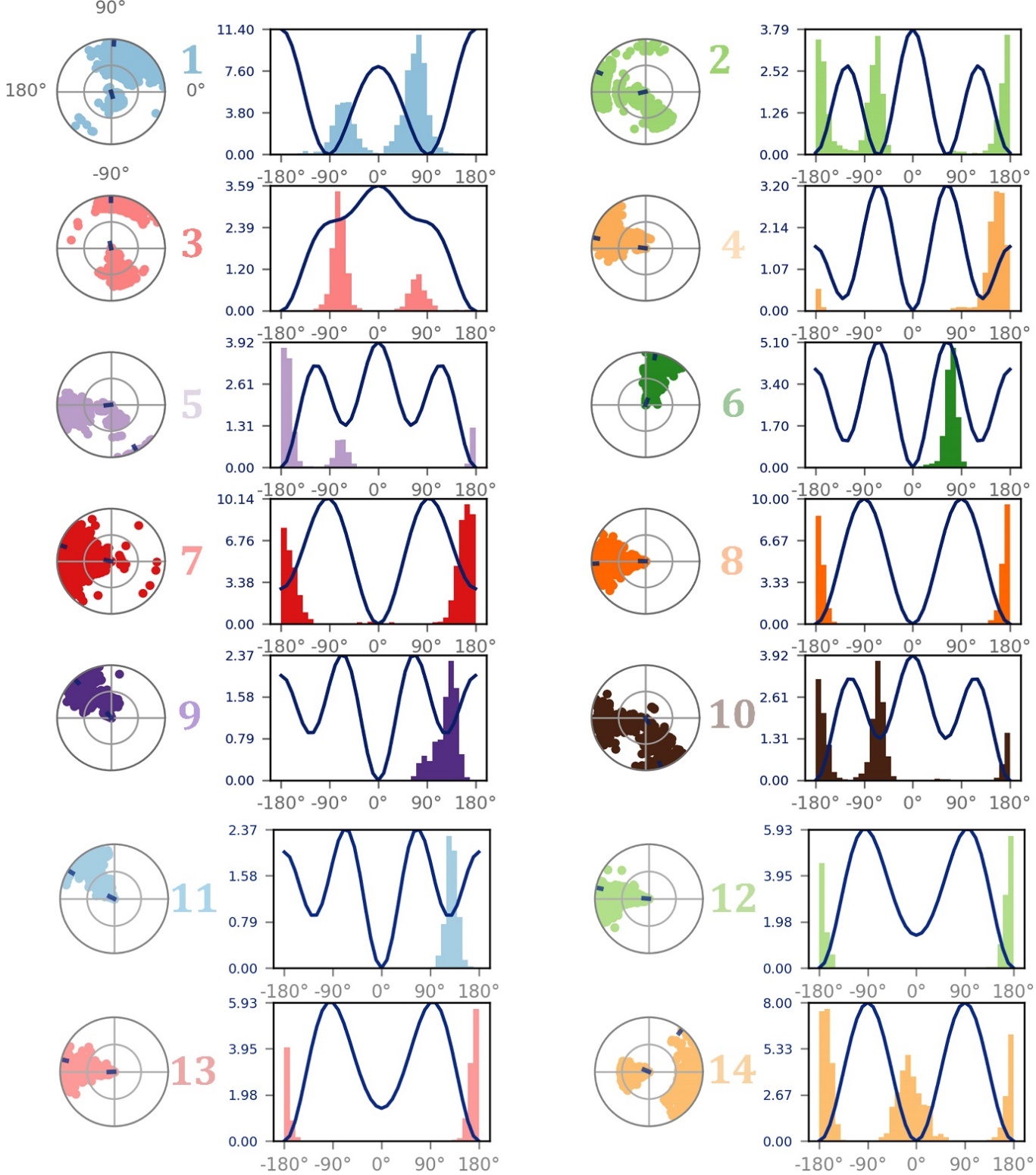


**Supplementary Figure 3:** Torsion angle graph ofAkuammicine N-Oxide at 100ns run of MD Simulation.
